# A CHCHD6–APP axis connects amyloid and mitochondrial pathology in Alzheimer’s disease

**DOI:** 10.1007/s00401-022-02499-0

**Published:** 2022-09-14

**Authors:** Yutong Shang, Xiaoyan Sun, Xiaoqin Chen, Quanqiu Wang, Evan J. Wang, Emiko Miller, Rong Xu, Andrew A. Pieper, Xin Qi

**Affiliations:** 1 Department of Physiology and Biophysics, Case Western Reserve University School of Medicine, 10900 Euclid Ave, E516, Cleveland, OH 44106-4970 USA; 2 Center for Artificial Intelligence in Drug Discovery, Case Western Reserve University School of Medicine, Cleveland, OH 44106 USA; 3 Beachwood High School, Beachwood, OH 44122 USA; 4 Harrington Discovery Institute, University Hospitals Cleveland Medical Center, Cleveland, OH 44106 USA; 5 Department of Psychiatry, Geriatric Research Education and Clinical Centers, Case Western Reserve University, Louis Stokes Cleveland VAMC, Cleveland, OH 44106 USA

**Keywords:** Alzheimer’s disease, Amyloid-beta precursor protein, Mitochondria, CHCHD6, Neuroprotection, Neurodegeneration

## Abstract

The mechanistic relationship between amyloid-beta precursor protein (APP) processing and mitochondrial dysfunction in Alzheimer’s disease (AD) has long eluded the field. Here, we report that coiled-coil-helix-coiled-coil-helix domain containing 6 (CHCHD6), a core protein of the mammalian mitochondrial contact site and cristae organizing system, mechanistically connects these AD features through a circular feedback loop that lowers CHCHD6 and raises APP processing. In cellular and animal AD models and human AD brains, the APP intracellular domain fragment inhibits CHCHD6 transcription by binding its promoter. CHCHD6 and APP bind and stabilize one another. Reduced CHCHD6 enhances APP accumulation on mitochondria-associated ER membranes and accelerates APP processing, and induces mitochondrial dysfunction and neuronal cholesterol accumulation, promoting amyloid pathology. Compensation for CHCHD6 loss in an AD mouse model reduces AD-associated neuropathology and cognitive impairment. Thus, CHCHD6 connects APP processing and mitochondrial dysfunction in AD. This provides a potential new therapeutic target for patients.

**Supplementary Information:**

The online version contains supplementary material available at 10.1007/s00401-022-02499-0.

## Introduction

Alzheimer’s disease (AD) is a progressive neurodegenerative disorder leading to dementia, for which there are currently no effective treatments. AD is characterized by progressive extracellular accumulation of amyloid-beta (Aβ) plaques, intracellular neurofibrillary tangles, synaptic loss, and cognitive impairment [25, 34]. While neurotoxic Aβ peptide produced via proteolysis of amyloid precursor protein (APP) is a major component of AD pathogenesis, other APP proteolytic fragments, such as C99 and amyloid precursor protein intracellular domain (AICD), also drive pathology [23, 26, 30, 50, 63]. Furthermore, missense APP mutations are associated with early-onset familial AD, and failure to clear APP-dependent Aβ accumulation from the brain is implicated in late-onset sporadic AD [15, 34]. APP is highly expressed in the brain and metabolized by sequential secretase activity, yet attempts to treat AD by interfering with this secretase activity have failed to help patients. Thus, exploring other aspects of APP biology, such as its interface with mitochondrial function, are important for understanding the disease and developing effective therapies.

Mitochondrial impairment is also a prominent and early feature of AD [46, 69], and is linked to amyloid biology by virtue of APP localization to the outer mitochondrial membrane (OMM), inner mitochondrial membrane (IMM), and mitochondrial matrix [13, 44]. APP is also highly enriched in mitochondria of the hippocampus and other brain regions that are particularly vulnerable in AD [7, 13], and APP-deficient mice exhibit extensively compromised mitochondrial bioenergetics [41]. APP overexpression in mice also causes mitochondrial fragmentation, respiratory failure, and neuronal apoptosis [10, 52, 58], and neurons with impaired mitochondrial function display aberrant APP processing [51, 72, 76]. Thus, a proper balance of APP is essential for healthy mitochondrial function. APP is processed in large part at mitochondria-associated endoplasmic reticulum (ER) membranes (MAM) [12], and accumulation of the C99 APP fragment at MAMs is associated with disrupted lipid homeostasis that leads to toxic brain cholesterol accumulation [37, 45]. Despite the known APP-mitochondrial interactions in AD, however, the underlying mechanism of this interaction has not previously been identified.

A crucial component of healthy mitochondrial function is the mitochondrial contact site and cristae organizing system (MICOS) multi-subunit protein complex, which is located in the IMM adjacent to the cristae junction (CJ). The MICOS maintains normal mitochondrial cristae morphology and homeostasis, and has been highly conserved in form and function throughout evolution [16, 27]. In mammals, MICOS consists of seven proteins, with coiled-coil-helix-coiled-coil-helix domain containing 6 (CHCHD6), mitofilin, and coiled-coil-helix-coiled-coil-helix domain containing 3 (CHCHD3) comprising the centerpiece of the large 700 kDa protein core complex [40]. An intact and properly functioning MICOS is required to stabilize the highly curved membrane of CJs, which allows them to form their normal narrow, neck-like structures [16, 57]. Notably, altered MICOS protein levels, mutations in MICOS protein-encoding genes, and MICOS protein post-translational modifications have all been linked to neurodegenerative disease [3, 56, 62, 64]. Furthermore, down-regulation of MICOS in yeast, *Drosophila*, and HeLa cells results in a disorganized IMM with closely packed stacks of membrane sheets replacing tubular or vesicular cristae [2, 31, 40, 66]. This disrupts mitochondrial bioenergetic function since cristae house the respiratory chain complexes. MICOS also acts as a diffusion barrier for proteins and other small molecules, which enables the internal space of cristae to be a stable micro-compartment that adapts for optimal mitochondrial function, including oxidative respiration [57, 73]. Thus, it is not surprising that aberrant mitochondrial architecture caused by MICOS disruption results in increased mitochondrial reactive oxygen species (ROS) production and cell death, concomitant with membrane depolarization and decreased production of adenosine triphopshate (ATP) [2, 31, 40, 66, 73]. MICOS also participates in lipid metabolism and mitochondrial protein import through specific interactions with IMM and OMM proteins [1, 16, 60].

Within the MICOS, CHCHD6 localizes at the peripheral IMM where it couples mitochondrial import to bioenergetic state [2, 14]. Genetic depletion of CHCHD6 significantly disrupts MICOS integrity, causing cristae loss and bioenergetic failure [2, 14, 16]. Reduced CHCHD6 also impairs glucose metabolism by prompting a shift from oxidative phosphorylation to glycolysis [9]. Importantly, recent large-scale proteomic analyses have revealed that CHCHD6 is significantly decreased in postmortem brain tissue from AD patients [5, 75]. However, the precise role of CHCHD6 in AD mitochondrial biology has not been determined. Here, we report that CHCHD6 interacts with APP to impact both amyloid and mitochondrial pathology. Specifically, CHCHD6 is selectively decreased in both in vitro and in vivo APP-based AD models, as well as AD patient brains. We show that CHCHD6 is transcriptionally down-regulated by AICD binding to the CHCHD6 promoter, in partnership with Fe65 and Tip60 cofactors. We further observe that APP and CHCHD6 physically interact and stabilize each other under normal healthy conditions. Loss of CHCHD6 in neuronal cells impairs this process and promotes APP accumulation on the MAM, which accelerates aberrant APP processing. We also show that CHCHD6 depletion in neuronal cells disrupts mitochondrial cristae integrity, impairs bioenergetic activity, and kills cells. In addition, downregulation of CHCHD6 in the hippocampus of APP-based mouse models of AD mice promotes aberrant neuronal cholesterol accumulation, amyloidogenesis, synaptic loss, and neuroinflammation, with compensation for CHCHD6 loss mitigating this pathology. Thus, we report here a previously unidentified CHCHD6–APP axis that for the first time mechanistically connects aberrant APP metabolism and impaired mitochondrial bioenergetics in AD, leading to neurodegeneration and impaired cognition. Our demonstration that compensation for CHCHD6 deficiency mitigates AD-associated neuropathology and cognitive impairment suggests that stabilization of the CHCHD6–APP axis represents a novel therapeutic target for AD patients.

## Methods

### Reagents and antibodies

Protein phosphatase inhibitor cocktail (P5726), protease inhibitor cocktails (8340), and filipin (F4767) were purchased from Sigma-Aldrich (St. Louis, MO, USA). The antibodies against ATPB (17247-1-AP, 1:2000), CHCHD6 (20639-1-AP, 1:2000), CHCHD3 (25625-1-AP, 1:1000), Tom20 (11802-1-AP, 1:4000), Lonp (15440-1-AP, 1:1000), SigmaR1 (15168-1-AP, 1:1000) and p62 (18420-1-AP, 1:1000) were from ProteinTech (Rosemont, IL, USA). The antibodies against FACL4 (sc-365230, 1:1000), c-Myc (sc-40, 1:1000), Tim23 (sc-514463, 1:1000) were purchased from Santa Cruz Biotechnology (Dallas, TX, USA). The antibodies against APP (ab32136, 1:5000), cytochrome C (ab110325, 1:10000), VDAC (ab14734, 1:2000), Mitofilin (ab110329, 1:500), synaptophysin (ab32127, 1:10000), and Fe65 (ab5668, 1:500) were from Abcam (Cambridge, UK). The antibodies against the C-terminal of APP (A8717, 1:5000), NeuN (A60, MAB377, 1:1000), FLAG (F3165, 1:2000), and β-actin (A1978, 1:10000) were obtained from Sigma-Aldrich. The Iba1 (019–19741, 1:1000) antibody was from Wako Chemicals (Japan). The antibodies against GFAP (MAB360, 1:1000) and NeuN (ABN90, 1:1000) were purchased from MilliporeSigma (Burlington, MA, USA). The PSD-95 antibody (MA1-045, 1:500) was obtained from Invitrogen (Waltham, MA, USA). The antibody against purified anti-β-amyloid 1–16 (clone 6E10, #803015, 1:2000), and AICD (811901, 1:200) and APP recombinant protein (PTN-5279) were from BioLegend (San Diego, CA, USA). The antibody against Tip60 (12058, 1:200) and LC3B (2775S,1:2000) was from Cell Signaling (Danvers, MA, USA). The HRP-conjugated anti-mouse and rabbit secondary antibodies were from Thermo Fisher Scientific. The VeriBlot secondary antibody (HRP) (ab131366, 1:2000), which does not recognize heavy or light chains, was from Abcam. The Alexa 488/568/405/633 fluorescent secondary antibodies were from Invitrogen. CHCHD3 (CHCHD3-195H) and Mitofilin (IMMT-838H) recombinant proteins were from Creative Biomart (Shirley, NY, USA). CHCHD6 recombinant protein (TP302975) was from Origene (Rockville, MD, USA). The information on the antibodies used in this study is listed in Supplementary Table 1.

### Cell culture

Mouse hippocampal HT-22 cells (Millipore, SCC129), human embryonic kidney–293 T cells (HEK293, ATCC, CRL-1573), and Neuro2a cells (ATCC, CCL-131) were cultured in Dulbecco’s modified Eagle’s medium (DMEM) supplemented with 10% (v/v) heat-inactivated FBS and 1% (v/v) antibiotics (100 unit/mL penicillin, 100 μg/mL streptomycin). Neuro2a cells stably overexpressing human APP wildtype (APP^wt^) or Swedish mutant (APP^swe^, K670N and M671L APP, clone Swe.10) were obtained from Dr. Gopal Thinakaran (University of Chicago) and maintained as described above. All cells were maintained at 37 °C and 5% CO_2_.

### Constructs and transfection

To construct the CHCHD6-Flag plasmid, mouse CHCHD6 was amplified from Neuro2a cDNA samples through standard PCR methods, and then cloned into pcDNA3.1 ( +)-Flag vector between EcoR1 and Xho1 restriction sites. For Fe65, AICD, and Tip60 overexpression in HEK293T cells, cells were transfected with Myc-DDK-hFe65 (Rockville, MD, USA, Origene, RC202003), pAAV-hAICD-NLS-IRES-hrGFP (Watertown, MA, USA, addgene, catalog no. 107543), and pCMV3-Tip60-Flag (Beijing, China, Sino Biological, HG17131-CF) plasmids using TransIT^®^-2020 transfection reagent (Mirus Bio LLC, Madison, WI), according to the manufacturer’s instructions. For knockdown of APP in HEK293T cells, cells were infected with lentivirus of control shRNA and APP shRNAs (Sigma, TRCN0000011043, TRCN0000006706 and TRCN0000006707). Lentiviruses were generated by transfecting HEK293T cells with plasmids that encoded the envelope (pCMV-VSV-G; catalog no. 8454, Addgene), packing (psPAX2; catalog no. 12260, Addgene), and targeted open reading frame. After 12 h of transfection, the medium was changed, and the lentiviruses were harvested after 36 h. The lentiviruses were diluted with the corresponding medium at a 1:1 ratio, and the cells of interest were infected in the presence of Polybrene (8 g/mL, Sigma-Aldrich) for 48 h.

To construct AAV-hSyn-CHCHD6-EGFP plasmid, mouse CHCHD6 was amplified from Neuro2a cDNA samples through standard PCR methods, and then cloned into control AAV plasmid backbone, pAAV.hSynapsin.EGFP.WPRE.bGH(Osten-Frank)(p1696)-Q (Cat# AV-5-PV1696) which was obtained from Penn Vector Core, University of Pennsylvania. To construct AAV-CHCHD6 shRNA-mCherry plasmid, CHCHD6 shRNA was amplified from mouse CHCHD6 shRNAs (Sigma, TRCN0000240966), and then inserted into pAAV-U6-hSyn.mCherry.3xFLAG-WPRE (addgene, 120392) after U6 promoter. pAAV-U6-Scrambled shRNA-hSyn::mCherry.3xFLAG-WPRE (addgene #120395) was used as control. AAV-GFP-CHCHD6, AAV-GFP control, AAV-mCherry-Scrambled shRNA and AAV-mCherry-CHCHD6 shRNA were then packed to obtain AAV5 particles by VectorBuilder (Guangzhou, China).

### CHCHD6 KO by CRISPR-Cas9

To silence CHCHD6 in HT-22 cells, a control and CHCHD6 synthetic guide RNA (sgRNA) CRISPR lenti-vector set (K3156005) was purchased from Applied Biological Materials (Richmond, BC, Canada). HT-22 cells were transfected as described with either control or CHCHD6 sgRNA. Stable HT-22 cells with CHCHD6 KO were selected using puromycin (Corning) at 5 μg/mL and maintained at 2 μg/mL.

### Preparation of oligomeric Aβ_1–42_

The Aβ_1–42_ (GenicBio Limited) peptides were dissolved in 1,1,1,3,3,3-hexafluoro-2-propanol (HFIP; Sigma-Aldrich) to a final concentration of 5 mM and placed in a chemical hood overnight. The next day, HFIP was further evaporated using a SpeedVac concentrator for 1 h. Monomer Aβ (5 mM) was prepared by dissolving Aβ peptide in anhydrous dimethyl sulfoxide (Sigma-Aldrich). The oligomeric Aβ peptides were prepared by diluting the monomer Aβ solution in Dulbecco’s Modified Eagle Medium (DMEM)/F12 and then incubating at 4 °C for 24 h.

### Animal model of AD

All animal experiments were conducted in accordance with protocols approved by the Institutional Animal Care and Use Committee of Case Western Reserve University and performed according to the National Institutes of Health Guide for the Care and Use of Laboratory Animals. Sufficient procedures were employed to reduce the pain and discomfort of the mice during the experiments. All mice were maintained under a 12 h/12 h light/dark cycle (light on at 6 AM and off at 6 PM) with ad libitum access to food and water under the ambient temperature at 23 °C and humidity at 40–60%. The mice were mated, bred, and genotyped in the animal facility of Case Western Reserve University. All mice used in this study were maintained on a C57BL/6J (Strain #000664, The Jackson Laboratory) background. 5XFAD transgenic mice [Tg(APPSwFlLon,PSEN1*M146L*L286V)6799Vas, strain #034840-JAX] breeders were purchased from Jackson Laboratory. APP^NL−G−F^ and APP^NL−F^ knock-in breeder mice (stock # RBRC06344 and # RBRC06343) were obtained from Japan Riken BioResource Research Center. All mice are genotyped before experiments based on the genotyping protocol the vendors provided.

### Stereotaxic injection

Stereotaxic surgery was performed using a model 1900 stereotax (Kopf) under isoflurane anesthesia. Briefly, a small craniotomy was made using a 33-gauge drill bit above the desired coordinate. A small pulled glass pipette containing AAV was attached to a Nanoject II (Drummond) and was then inserted to the appropriate depth. Injections were performed at a rate of 90 nl/min. The coordinates used for bilaterally hippocampus injections were anteroposterior, − 2.0 mm from bregma; mediolateral, ± 2.0 mm; dorsoventral, − 1.7 mm). AAV5-GFP control and AAV5-GFP-CHCHD6 were injected into the hippocampus of APP^NL−G−F^ Knock-in mice and wild-type littermates at the age of 3 months for CHCHD6 overexpression. AAV5-mCherry-Scrambled shRNA and AAV5-mCherry-CHCHD6 shRNA were injected into the hippocampus of APP^NL−F^ Knock-in mice and wild-type littermates at the age of 6 months for CHCHD6 knocking down. The concentration (ddTiter) of the AAVs is 1.0e12 GC/mL. 1 μL was injected into each hemisphere of mice. As a result, the number of viral particles of AAVs is 1.0e9.

### Behavioral analysis

All behavioral analyses were conducted by an experimenter who was blinded to the genotypes and treatment groups. All mice were subjected to a series of behavioral measurements to monitor spontaneous spatial working memory (Y-maze test) and long-term spatial learning, and memory functions (Barnes maze test). Body weights was recorded throughout the study period.

#### Y-maze test

On the test day, mice were brought to the testing room one hour before performing the Y-maze test to allow habituation. The mice were placed in the middle of the Y-maze and allowed to explore the three arms for 6 min. During exploration, the arm entries were recorded. The equipment was cleaned after every test to avoid odor disturbance. Spontaneous alternation was defined as a successive entry into three different arms on overlapping triplet sets. APP^NL−G−F^ knock-in mice and age-matched wild-type littermates injected with AAV5-GFP control and AAV5-GFP-CHCHD6 were tested at 6 and 9 months old. APP^NL−F^ knock-in mice and age-matched wild-type littermates injected with AAV5-mCherry-Scrambled shRNA and AAV5-mCherry-CHCHD6 shRNA were tested at 9 and 12 months old.

#### Barnes maze test

On the test day, the mice were brought to the testing room 1 h before performing the Barnes maze test to allow habituation. Briefly, all the testing mice received three consecutive days of trials, with three trials each day. After being placed in the center of the platform at the beginning of each trial, the mice were allowed to explore for 3 min to find the target escape box. Mice that failed to enter the target escape hole in the given time were led to it by the operator. Mice were allowed to remain in the target hole for 2 min before returning to the home cage. After completing the 3-day trials, the mice were examined on days 5 and 12 with one test to monitor the long-term spatial learning and memory activities. The maze and the escape box were cleaned carefully after each trial to avoid odor disturbance. All the trials and tests were recorded with a video system. The total time to enter the target escape box (latency to the target box) and the number of times the wrong holes were explored (the total errors) were recorded. APP^NL−G−F^ knock-in mice and age-matched wild-type littermates injected with AAV5-GFP control and AAV5-GFP-CHCHD6 were tested at 9 months old. APP^NL−F^ knock-in mice and age-matched wild-type littermates injected with AAV5-mCherry-Scrambled shRNA and AAV5-mCherry-CHCHD6 shRNA 6 were tested at 12 months old.

### Preparation of total lysates

Cells were washed in cold PBS (pH 7.4) and incubated on ice for 30 min in total lysis buffer (50 mM Tris–HCl, pH 7.5, 150 mM NaCl, 1% Triton X-100, protease inhibitors cocktail, and phosphatase inhibitors cocktail (MilliporeSigma)). Mouse brains were minced and homogenized in lysis buffer and placed on ice for 30 min. Cells or tissues were centrifuged at 12,000 × *g* for 10 min at 4 °C to generate total lysate supernatants.

### Isolation of mitochondria-enriched fractionations

Cells were washed with cold PBS and incubated on ice in mitochondrial lysis buffer (250 mM sucrose, 20 mM HEPES–NaOH, pH 7.5, 10 mM KCl, 1.5 mM MgCl2, 1 mM EDTA, 1 mM EGTA, protease inhibitor cocktail, and phosphatase inhibitor cocktail) for 30 min. The cells were scraped and then disrupted 20 times by repeated aspiration through a 25-gauge needle. Mouse brains were minced and homogenized as described above in mitochondrial lysis buffer to release mitochondria. The homogenates were spun at 800 × g for 10 min at 4 °C, and the resulting supernatants were spun at 10,000 × *g* for 20 min at 4 °C. The pellets were washed with mitochondrial lysis buffer and spun at 10,000 × *g* again for 20 min at 4 °C. The final pellets of mitochondria were suspended in lysis buffer containing 1% Triton X-100 and were assigned as mitochondrial-rich lysate fractions. For native PAGE detecting protein complexes, mitochondria pellets were incubated in 1 × NativePAGE Sample Buffer (NativePAGE™ Sample Prep Kit, Thermo Fisher, BN2008) containing 2% Digitonin (sigma, D141), and incubated on ice for 30 min, and then centrifuged at 20,000 × *g* for 30 min at 4 °C to generate mitochondrial-rich lysate supernatants.

### Mitochondrial sub-compartmental fractionation

Isolation of mitochondrial sub-compartmental fractions, including the ER, mitochondria, and ER–mitochondrial-associated membranes, was performed as previously described [70]. Briefly, HT-22 control and CHCHD6 KO cells were washed with cold PBS and detached from the plates using scraper. The cells were collected by centrifugation at 600 × *g* for 5 min then resuspended in ice-cold mitochondrial isolation buffer (225 mM mannitol, 75 mM sucrose, 0.1 mM EGTA and 30 mM Tris–HCl, pH 7.4). The cells were homogenized by repeated aspiration through a 25-gauge needle. Then, cell debris and nuclei were removed from the homogenate by centrifugation, and the supernatants were subjected to further differential centrifugation to get the crude mitochondrial fraction in the pellet. The supernatants were further centrifuged at 100,000 × *g* for 1 h to obtain the ER fraction. The crude mitochondrial fraction was resuspended in mitochondrial resuspending buffer (MRB, 250 mM mannitol, 0.5 mM EGTA, and 5 mM HEPES, pH 7.4) to the final volume of 2 mL, and the crude mitochondrial suspension was layered on the top of the Percoll medium (225 mM mannitol, 25 mM HEPES pH 7.4, 1 mM EGTA and 30% Percoll (vol/vol)). The pure mitochondrial and MAM fractions were isolated by centrifugation at 95,000 × *g* for 30 min, washed to remove the Percoll, and further purified by centrifugation to eliminate contaminants. All fractions were reconstituted in mitochondrial resuspending buffer and stored at − 80 °C until analysis.

### Western blotting

#### SDS-PAGE

The protein concentration in each sample was determined using protein assay dye reagents (Bio-Rad, 5000006). The proteins were resuspended in Laemmli buffer, separated using SDS gels, and transferred to nitrocellulose membranes. The membranes were probed with the indicated antibodies, and the specific proteins were visualized by electrochemiluminescence.

#### BN-PAGE

2% digitonin extracted protein complexes from mitochondria purified from cells or mice brain tissues were resuspended in NativePAGE 5% G-250 Sample Additive (Invitrogen, BN2004), and then separated by blue native polyacrylamide gel electrophoresis (BN-PAGE) on a precast native 4–16% Bis–Tris Protein Gels (Thermo Fisher, BN1002BOX).

### Total RNA isolation and real-time quantitative RT-PCR

Total RNA was isolated using the RNeasy Mini Kit (QIAGEN, Hilden, Germany, 74104) or TRIzol Reagent (15596-026, Invitrogen), and cDNA was synthesized from 1 μg of total RNA using the QuantiTect Reverse Transcription Kit (QIAGEN, 205311). qRT-PCR was performed with SYBR Green mix (Thermo Fisher Scientific, A25743) and analyzed using the StepOnePlus Real-Time PCR System (Thermo Fisher Scientific). At least two replicates were performed with each biological sample, and the expression values of each replicate were normalized against GAPDH cDNA using the 2-ΔΔCT method. The primers used in this study are presented in Supplementary Table 2.

### Immunofluorescence

Cells were grown on coverslips, fixed with 4% paraformaldehyde for 20 min at room temperature, permeabilized with 0.1% Triton X-100 in PBS, and blocked with 2% normal goat serum. The cells were incubated with the indicated primary antibodies overnight at 4 ℃. After washing with PBS, the cells were incubated with Alexa Fluor goat anti-mouse/rabbit 488 or 568 secondary antibody (1:1000; Thermo Fisher Scientific) for 2 h at room temperature. The nuclei were counterstained with Hoechst dye (1:10,000; Sigma-Aldrich). Coverslips were mounted and slides were imaged by a confocal microscope (Olympus, Tokyo, Japan; Fluoview FV100).

For immunofluorescence staining of mouse brain sections, mice were deeply anesthetized and transcardially perfused with 4% paraformaldehyde in PBS. Brain sections were permeabilized with 0.2% Triton X-100 in TBS-T buffer, followed by blocking with 5% normal goat serum. The brain sections were incubated with the indicated primary antibodies overnight at 4 °C and then stained with 488/568/405/633 secondary antibodies (1:500). Images of the staining were acquired using a confocal microscope (Olympus).

For detection of cholesterol, brain sections were stained with filipin (F4767, Sigma-Aldrich) at room temperature in the dark. Filipin staining was imaged using an all-in-one fluorescence microscope (Keyence).

All quantification of immunostaining was performed using ImageJ software. The same image exposure times and threshold settings were used for all sections from all the experimental groups. Quantitation was performed blinded to the experimental groups.

### MMP measurements

HT-22 cells cultured on coverslips were washed in PBS (pH 7.4) and incubated with 0.25 μM TMRM and 5 μg/mL Hoechst for 20 min at 37 °C. Images were visualized by confocal microscopy (Fluoview FV1000, Olympus) and image quantification was performed using the ImageJ software. At least 90 cells/group were counted for analysis. TMRM fluorescence density was normalized to the total number of cells.

### Measurement of mitochondrial respiratory capacity

Mitochondrial respiration activity in HT-22 EV and CHCHD6 KO cells was analyzed using a Seahorse Bioscience XFp Extracellular Flux Analyzer (Agilent). Briefly, cells were seeded in XFp 8-well miniplates (103,025–100, Agilent, Santa Clara, CA, USA) at 8000 cells/well in 100 μL growth medium. One hour prior to measuring oxygen consumption, the cell culture media was replaced with XF assay medium (1 mM pyruvate, 2 mM glutamine, and 10 mM glucose in basal DMEM) and maintained in a non-CO2 incubator for 1 h. The sensor cartridges were placed in the XFp Analyzer according to the manufacturer’s instructions for the Mito Stress Test kit (Agilent, 103,010–100). Mitochondrial function was determined by the sequential injection of oligomycin A (1 μM), FCCP (1 μM), and rotenone/antimycin A (0.5 μM). Following each experiment, the total cellular number of each well was determined using DAPI staining.

### Determination of total ATP level

The cellular ATP concentration was measured using an ATP Colorimetric/Fluorometric Assay Kit (BioVision, K354). Neuro2a control cells and APP-overexpressing stable lines (APP^wt^ and APP^swe^) were transfected with Flag-EV or Flag-CHCHD6 plasmids for 48 h. Then, cells were lysed in 50 μL of ATP assay buffer, and total ATP level was determined at 570 nm using a microplate reader, according to the manufacturer’s instructions.

### Measurement of cell viability

HT-22 EV and CHCHD6 KO cell lines were transfected with Flag-EV or Flag-CHCHD6 plasmids. After 24 h transfection, cells were treated with oligomeric Aβ peptides (5 µM) for 48 h. Cell viability was measured using an in vitro toxicology assay, MTT-based kit (Roche, 11465007001), according to the manufacturer’s instructions.

### RNA-Seq and bioinformatics

Total RNA of HT-22 EV and CHCHD6 KO cells were purified using RNeasy mini kit (Qiagen). For each sample, 200 ng RNA was used as templates. RNA-Seq was processed by Thermo Fisher using the Clariom S Assay, mouse. The data were analyzed by Transcriptome Analysis Console (TAC) 4.0.2 and the pathway analysis was carried out with g:Profiler server (https://biit.cs.ut.ee/gprofiler/convert).

### Total cholesterol measurements by ELISA

The total cholesterol content was measured using the total cholesterol assay kit (STA-384, Cell Biolabs), according to the manufacturer’s instructions. Briefly, the total lipids were extracted from hippocampus brain tissue samples using a mixture of chloroform, isopropanol, and NP-40 (7:11:0.1). The homogenates were centrifuged at 15,000 × *g* for 10 min, and the supernatants were collected and dried to remove the organic solvent. The dried lipids were dissolved in 1 × assay diluent for further quantification assay by adding cholesterol reaction reagent (cholesterol oxidase 1:50, HRP 1:50, colorimetric probe 1:50 and cholesterol esterase 1:250 in 1 × assay diluent). The calculated amount of total cholesterol for each sample was normalized to the weight of the hippocampus tissue.

### In situ proximity ligation assay (PLA)

PLA was performed using the Duolink^®^ In Situ Red Starter Kit (mouse/rabbit, DUO92101, Sigma), and Duolink In Situ Detection Reagents Green (DUO92014, Sigma) as described previously [76]. Briefly, fixed cells or brain sections were permeabilized and blocked with PLA blocking buffer for 1 h at 37 °C and incubated with the indicated primary antibodies overnight at 4 °C. The samples were then incubated with the PLA probes (Anti-Rabbit PLUS and Anti-Mouse MINUS) for 1 h at 37 °C, followed by the ligation and amplification steps. The PLA signal was visible as a distinct fluorescent spot and analyzed by confocal microscopy (Fluoview FV1000, Olympus). The number of fluorescent signals was quantitated using NIH ImageJ software.

### Luciferase assay

5’-Flanking region of the human CHCHD6 gene was amplified by PCR using genomic DNA from HEK293 cells as templates. After amplification, PCR products were inserted into the pGL3-luciferase vector (Promega, Madison, WI). HEK293 cells were transfected with the appropriate cDNAs: empty vector (pGL3-luc), CHCHD6 promoter-luc, AICD, and/or Fe65, and/or Tip60. 36 h after transfection, cells were rinsed, gently scraped into PBS (pH 7.4), and pelleted. Cells were then lysed in lysis buffer, and the luciferase activity was measured by the Luciferase Assay System following the manufacturer's instructions (Promega, E1910).

### Chromatin immunoprecipitation (ChIP)

ChIP assays were performed using a chromatin immunoprecipitation (ChIP) assay kit (Millipore, 17–295) according to the manufacturer's instructions. Briefly, 1% formaldehyde was added directly to HEK293 cells to cross-link proteins to DNA. The cell pellet was lysed and sonicated. After centrifugation, the supernatant was incubated overnight at 4 °C with antibodies against APP C terminus (Abcam, ab32136), Fe65 (Abcam, ab5668), Tip60 (Cell Signaling, #12058) and normal rabbit IgG. After immunoprecipitation using protein A and G beads, the antibody/protein/DNA complex was incubated at 65 °C for 4 h to reverse the protein/DNA cross-links. The DNA was purified using PCR Purification kit (Qiagen) and used as a template for PCR amplification. Different pairs of CHCHD6 promoter primers were used for amplification (Supplementary Table 2). PCR products were resolved on 2% agarose gels and visualized after ethidium bromide staining.

### Immunoprecipitation

Total protein lysates were harvest from cells as described above. Cell lysates were incubated with the indicated primary antibodies or control IgG (sc-2025, Santa Cruz Biotechnology) overnight at 4 °C, followed by incubation with protein A/G beads (sc-2003, Santa Cruz Biotechnology) for 2 h at 4 °C. The immunoprecipitates were washed with total lysis buffer three times for a total of 30 min and then subject to WB analysis.

Recombinant proteins were mixed and incubated in buffer containing 50 mM Tris [pH 7.5] and 2.5 mM MgCl_2_ and 0.1% Triton. The mixture of recombinant proteins was IP with indicated antibodies as described above.

### Human postmortem brain samples

All postmortem brain samples were collected by the National Institutes of Health (NIH) NeuroBioBank (NBB; https://neurobiobank.nih.gov/) under the approval of the Institutional Review Boards (IRB) and the institution’s Research Ethics Board. All brains were donated to the NBB by informed consent through the Brain and Tissue Repositories sites. All brain specimens donated to the NIH NBB were assessed and reviewed by board-certified neuropathologists. A standard assessment was performed to document possible neuropathologies and establish a disease condition diagnosis. In addition, postmortem blood was sampled and submitted for serology and toxicology testing. The human postmortem brain samples used in the experiments were obtained from the NBB under a material transfer agreement (MTA) between the NIH and Case Western Reserve University, and listed in Supplementary Table 3.

### Computational analysis on the relevance of CHCHD6 with AD

The overall experiment consisted of (1) network construction: an integrated network of gene–pathway–phenotype–disease (GPPDN) was constructed from multiple data resources to model and capture the complex and heterogeneous interrelationships among genes, pathways, phenotypes and human diseases; (2) network-based prioritization: genes, pathways, human diseases and phenotypes were prioritized from GPPDN based on their relevance to the input CHCHD6.

### Network construction

The foundation of the knowledge-driven discovery system for CHCHD6 is GPPDN that comprised 7 context-sensitive semantic knowledge networks. GPPDN captures a large amount of semantic knowledge of tens of thousands of biomedical entities (nodes) and their relationships (edges).

*Disease–gene network from OMIM (omimDGN)*: omimGN is an unweighted directional network consisting of 5983 disease nodes, 8831 gene nodes and 15,462 edges (disease–gene pairs). We constructed omimGN from The Online Mendelian Inheritance in Man database, a comprehensive source of disease genetics.

*Disease–gene network from ClinVar (clinvarDGN)*: clinvarGN is an unweighted directional network consisting of 5842 diseases/phenotypes nodes, 4153 gene nodes, and 6884 edges. The network was constructed directly from ClinVar, a database of the relationships among human variations and phenotypes.

*Disease–gene network from GWAS (gwasDGN)*: gwasGN is an unweighted directional network consisting of 3422 nodes of common complex diseases, 18,532 gene nodes, and 98,218 edges. We constructed the network directly from The Catalog of Published Genome-Wide Association Studies (GWAS catalog), an exhaustive source of disease–gene associations from GWAS studies.

*Pathway–gene network (PathGN)*: PathGN is an unweighted directional network consisting of 8868 gene nodes, 1329 pathway nodes and 66,293 edges and was construct the network from the Molecular Signatures Database, a comprehensive resource of annotated pathways and gene sets.

*Protein–protein interaction network (PPIN)*: PPIN is weighted unidirectional network consisting of 22,982 gene nodes and 382,256 edges and was constructed the network directly from BioGrid, an online biological interaction repository with data compiled through comprehensive curation efforts.

*Mutational phenotype–gene network (PhenGN)*: PhenGN was constructed from The Mouse Genome Database (MGD), which contains large amounts of phenotypic descriptions of systematic genetic knockouts in mouse models. A total of 517,381 mutational/causal phenotype–gene annotations (9982 phenotypes and 11,021 mapped human genes) were obtained from MGD. PhenGN allows us to interrogate causal relationships between CHCHD6 and AD-related phenotypes. PhenGN is unweighted (same weights for all phenotype–gene connections on the network) and consisted of 21,003 nodes (9982 phenotype nodes, 11,021 gene nodes) and 517,381 edges.

*Links between MGN, PhenGN, PathGN, and PPIN*: these subnetworks were connected through common nodes. For example, MGN are linked to PhenGN network through shared gene nodes.

### Network prioritization

The goal of this study is to identify genes, pathways, phenotypes and diseases that are associated with CHCHD6 at the genetic, functional, phenotypic and disease levels. For the input gene “CHCHD6”, the algorithm prioritized genes, pathways, phenotypes and diseases from GPPDN based on the context-sensitive network-based ranking algorithm that we previously developed [11, 78]. The random walk-based approach is briefly described below. The movements of a random walker between any two sub-networks were regulated with jumping probabilities ${\lambda }_{{N}_{i}{N}_{j}}$ (${N}_{i}$ and ${N}_{j}$ can be any of the four sub-networks). For example, if a random walker stands on a gene node on *MGN*, which is connected with both *omimDGN*, *PathGN* and *PPIN*, it has the option to walk to *omimDGN* with the probability ${\lambda }_{12}$, to *PathGN* with the probability ${\lambda }_{13,}$ to *PPIN* with the probability ${\lambda }_{14}$ or stay within $omDGN$ with the probability ${1-\lambda }_{12}-{\lambda }_{13}-{\lambda }_{14}$. Given the seed node(s)/inputs, the ranking score for each node is iteratively updated by:

1 $$S_{k + 1} = \alpha M^{T} S_{k} + \left( {1 - \alpha } \right)S_{0} ,$$

${S}_{k+1}$ is the score vector at step *k* + 1, ${S}_{0}$ is the initial vector, and $1-\alpha$ is the restarting probability, *M* is the transition matrix. The transition matrix *M* was calculated as follows in (2)–(3):

2 $$M = \left[ {\begin{array}{*{20}c} {M_{1} } & {M_{1.} } & {M_{1n} } \\ {M_{m1} } & {M_{m} } & {M_{mn} } \\ {M_{n1} } & {M_{n.} } & {M_{n} } \\ \end{array} } \right]$$

*M* consisted of 49 (7*7) sub-matrices, each contains the transition probabilities within or between 7 sub-networks. Each sub-matrix was calculated by normalizing the rows in the adjacency matrix of the corresponding sub-networks using the jumping probabilities. Specifically, the off-diagonal sub-matrices corresponded to the bipartite networks that connected each two networks. These sub-matrices were calculated by first normalizing the rows of the bipartite network ${A}_{{N}_{i}{N}_{j}}$, and then weighing each row by the jumping probability ${\lambda }_{{N}_{i}{N}_{j}}$:

3 $$\left( {M_{{N_{i} N_{j} }} } \right)_{{kl}} = ~\left\{ {\begin{array}{*{20}c} {\lambda _{{N_{i} N_{j} }} \left( {A_{{N_{i} N_{j} }} } \right)_{{kl}} /\mathop \sum \limits_{l} \left( {A_{{N_{i} N_{j} }} } \right)_{{kl}} ~~~~~\mathop \sum \limits_{l} \left( {A_{{N_{i} N_{j} }} } \right)_{{kl}} ~ \ne 0} \\ {0~~~~~~~~~~~~~~~~~~~~~~~~~~~~~~~~~~~~~~~~~~~~~~otherwise} \\ \end{array} } \right.~~.$$

The diagonal submatrices corresponded to the transition probabilities within each one of the 7 sub-networks and were calculated by first normalizing the rows of adjacency matrix for $omimDGN, gwasDGN, ClinVarDGN, PhenGN, PathGN, PPIN, ChemicalGN$, and then weighing the rows by the probability of staying in the same network:

4 $$\left( {M_{{N_{i} }} } \right)_{kl} = \left( {1 - \sum I_{{N_{j} }} \lambda_{{N_{i} N_{j} }} } \right)\left( {A_{{N_{i} }} } \right)_{kl} /\mathop \sum \limits_{l} \left( {A_{{N_{i} }} } \right)_{kl} .$$

In (4), ${A}_{{N}_{i}}$ is the adjacency matrix of the submatrix ${N}_{i}$, and ${I}_{{N}_{j}}$ is an indicator function, whose value is 1 if the *kth* row of ${A}_{{N}_{i}{N}_{j}}$ contains at least one non-zero element. The output from the context-sensitive network-based algorithm was a list of genes, pathways, phenotypes and diseases prioritized based on their genetic, functional, phenotypic relevance to CHCHD6.

### Quantification and statistical analysis

Sample sizes were determined by a power analysis based on pilot data collected in our laboratory or from published studies. For animal studies, we used *n* = 10–15 mice/group for behavioral tests, *n* = 3–6 mice/group for biochemical analyses, and *n* = 3–6 mice/group for pathology studies. In cell culture studies, each experiment was independently conducted at least three times. For animal studies, we ensured randomization and blinded evaluations. For imaging studies, a blinded observer performed quantification analyses. No samples or animals were excluded from our analysis.

Data were analyzed using GraphPad Prism 8.0 (GraphPad Software, San Diego, CA, USA). Unpaired Student’s *t* test was used for comparisons between two groups. Comparisons between three or more independent groups were performed using one-way ANOVA, followed by Tukey’s post hoc test. Comparisons of the effect of independent variables on a response variable were performed using two-way ANOVA. All values are reported as mean ± standard error of the mean (SEM). Data are representative of at least three independent experiments. Statistical parameters are presented in each figure legend. We considered *p* < 0.05 as statistically significant.

## Results

### Decreased CHCHD6 in AD models is specifically associated with MICOS loss

**Fig. 1:**
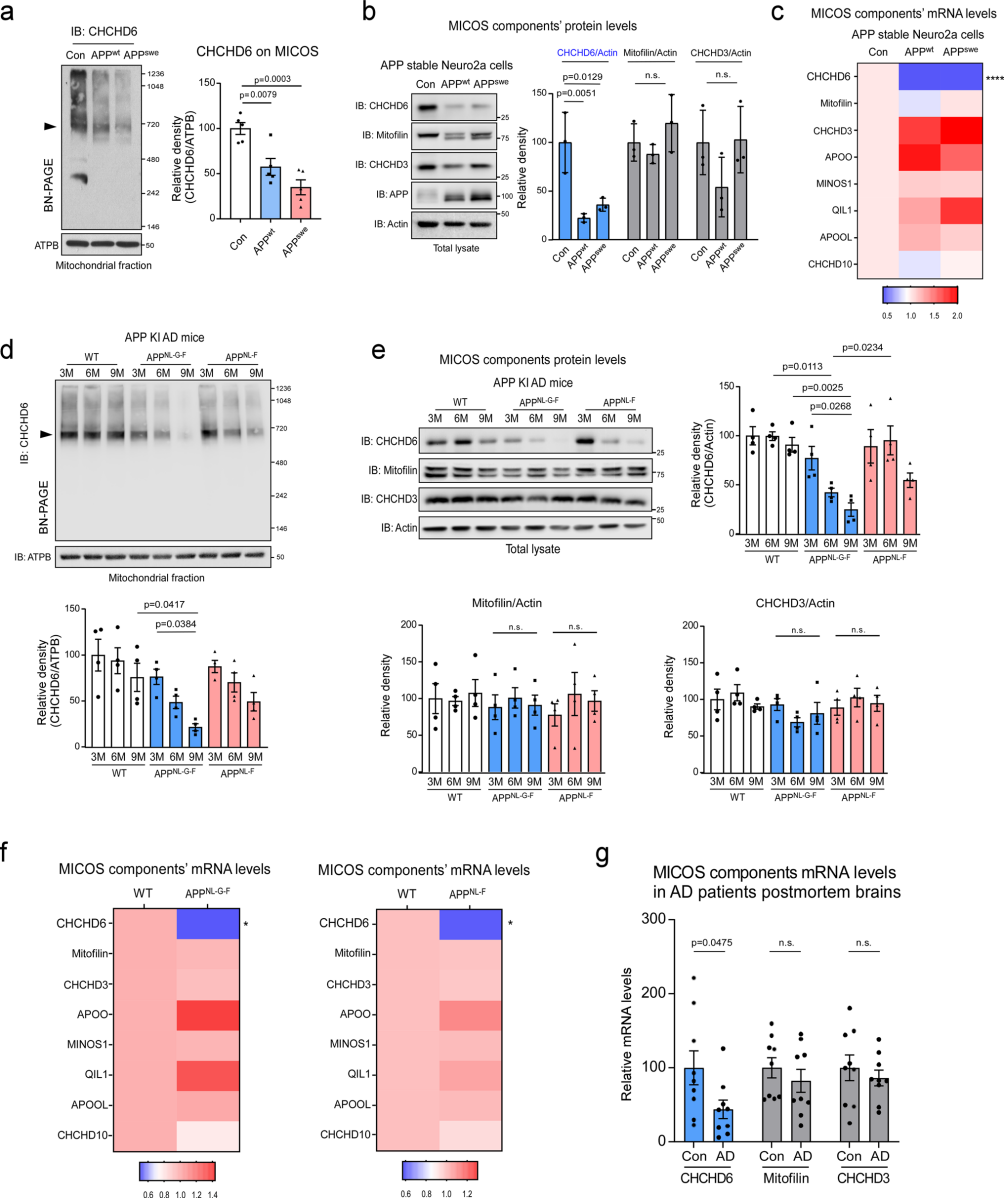
CHCHD6 selectively decreases at transcription level in AD models. **a** Mitochondrial fractions were isolated from APP stable Neuro2a cells and subjected to Blue Native PAGE (BN-PAGE) analysis followed by western blotting (WB). MICOS complex level was detected with antibody against CHCHD6. ATPB was analyzed by SDS-PAGE followed by WB, using as a loading control. Left: representative blots from 5 independent experiments. Right: relative density of CHCHD6-immunoreactive band around 720 kDa in contrast to ATPB. **b** Total lysates harvested from APP stable Neuro2a cells were subjected to WB with the indicated antibodies. Left: representative blots from 3 independent experiments. Right: relative density of CHCHD6, Mitofilin and CHCHD3 in contrast to actin. **c** RNA was extracted from APP stable Neuro2a cells. The expression of MICOS complex components was analyzed by qPCR. Heat map analysis shows the mean of the genes analyzed. *n* = 6 independent experiments for CHCHD10, and *n* = 4 independent experiments for others. ****p < 0.0001 (Con vs. APP^wt^ or APP^swe^ cells). **d** Mitochondrial fractions were isolated from the hippocampus of WT, APP^NL−G−F^ and APP^NL−F^ mice at the ages of 3, 6, and 9 months, and subjected to BN-PAGE analysis. Histogram: relative density of CHCHD6-immunoreactive band around 720 kDa in contrast to ATPB. *n* = 4 mice/group. **e** The hippocampus of WT, APP^NL−G−F^ and APP^NL−F^ mice was harvested at the ages of 3, 6, and 9 months. Total protein levels of CHCHD6, Mitofilin and CHCHD3 were examined by WB. *n* = 4 mice/group. **f** RNA was extracted from the hippocampus of 6-month-old APP^NL−G−F^ mice or 9-month-old APP^NL−F^ mice and age-matched wild-type (WT) mice. The expression of MICOS complex components was analyzed by qPCR. Heat map analysis shows the mean of the genes analyzed. *n* = 9 mice/group for CHCHD3 of APP^NL−G−F^ mice, and *n* = 5 mice/group for other groups. *p < 0.05 (WT vs. APP^NL−G−F^ mice or APP^NL−F^ mice). **g** RNA was extracted from the postmortem hippocampus of AD patients and control subjects. The expression of CHCHD6, Mitofilin and CHCHD3 was analyzed by qPCR. *n* = 9 individuals/group. All data are presented as mean ± SEM and were compared using one-way ANOVA with Tukey’s post hoc test in (**a**–**e**), and unpaired Student’s *t* test in (**f**–**g**)

In mammals, CHCHD6 complexes with mitofilin and CHCHD3 to form the ~ 700 kDa core component of MICOS, which governs mitochondrial membrane structural integrity and biogenesis [40]. The core component of MICOS is routinely detected by western blotting with antibodies against CHCHD6, mitofilin or CHCHD3, following blue native polyacrylamide gel electrophoresis (BN-PAGE) [19]. We applied this technique to determine whether the core component of MICOS is altered in cellular models of AD. Through BN-PAGE of isolated mitochondria from wildtype Neuro2a cells (Con), and Neuro2a cells that had been engineered to stably express either wildtype APP (APP^wt^) or the APP Swedish mutation associated with AD (APP^swe^) [61], we observed APP-dependent decreased CHCHD6 within the MICOS (Fig. 1a). The protein levels of mitofilin on the MICOS across all groups were unchanged (Supplementary Fig. 1a). This suggested CHCHD6-dependent disassembly of the MICOS complex. Western blot analysis of total protein lysates confirmed selective loss of CHCHD6, as mitofilin and CHCHD3 were both unaffected (Fig. 1b). Furthermore, quantitative real-time PCR (qPCR) revealed that CHCHD6 mRNA in both APP^wt^ and APP^swe^ cells was decreased by ~ 70% relative to Con (Fig. 1c). By contrast, mRNA levels of other components of the MICOS complex, including mitofilin, CHCHD3, MINOS1, apolipoprotein O (APOO), apolipoprotein O like (APOOL), CHCHD10 and QIL1 [29], were equivalent across all groups (Fig. 1c). Taken together, these results suggested to us that transcriptionally decreased levels of CHCHD6 could play an important role in dissolution of the MICOS core complex in AD.

We wondered whether a similar change in composition of the MICOS core complex also occurred in AD-model mice and human AD tissue. To address this question, we began with APP^NL−G−F^ and APP^NL−F^ knock-in (KI) mice. These mice carry human AD mutations in a humanized mouse APP locus driven by its endogenous promoter, and they develop amyloid plaques and neurocognitive defects [54]. The APP^NL−G−F^ KI construct contains a humanized Aβ region along with three pathogenic mutations, the Swedish “NL,” the Iberian “F,” and the Arctic mutation “G,” whereas the APP^NL−F^ KI construct only has the NL and F mutations [54]. Both lines of mice express APP at wild-type levels, avoiding possible artifacts introduced by APP overexpression while also producing elevated pathogenic Aβ [54]. The more extensively mutated APP^NL−G−F^ KI mice rapidly develop AD pathology and cognitive deficits, whereas APP^NL−F^ KI mice exhibit slower and more chronic disease progression [36, 54]. BN-PAGE of mitochondrial protein lysates from the hippocampus of APP KI or WT mice at 3, 6 and 9 months of age, followed by western blotting for MICOS core proteins, showed AD-specific and age-related decrease in hippocampal CHCHD6 on the MICOS, which was absent in WT mice and most severe in rapidly disease developing APP^NL−G−F^ KI mice (Fig. 1d). The level of mitofilin on the MICOS of these AD mice was not altered (Supplementary Fig. 1b). Consistent with our cell culture results (Fig. 1b), CHCHD6 was the only MICOS core complex protein found to be down-regulated with age in APP KI mice, and mitofilin and CHCHD3 levels remained comparable across APP KI and WT mice of all ages (Fig. 1e). The decrease in CHCHD6 occurred earlier and with greater magnitude in rapidly disease developing APP^NL−G−F^ KI mice, relative to chronically disease developing APP^NL−F^ KI mice (Fig. 1e). Notably, hippocampal CHCHD6 mRNA was also significantly decreased in both APP KI mice and 5XFAD mice that overexpress APP Swedish, Florida and London mutations (Fig. 1f, Supplementary Fig. 1c). Furthermore, the same decrease was seen in postmortem hippocampus of AD patients (Fig. 1g). Importantly, and consistent with our findings from cellular and animal AD models, the levels of mRNA for mitofilin and CHCHD3 were not altered in human AD hippocampus (Fig. 1g).

**Fig. 2:**
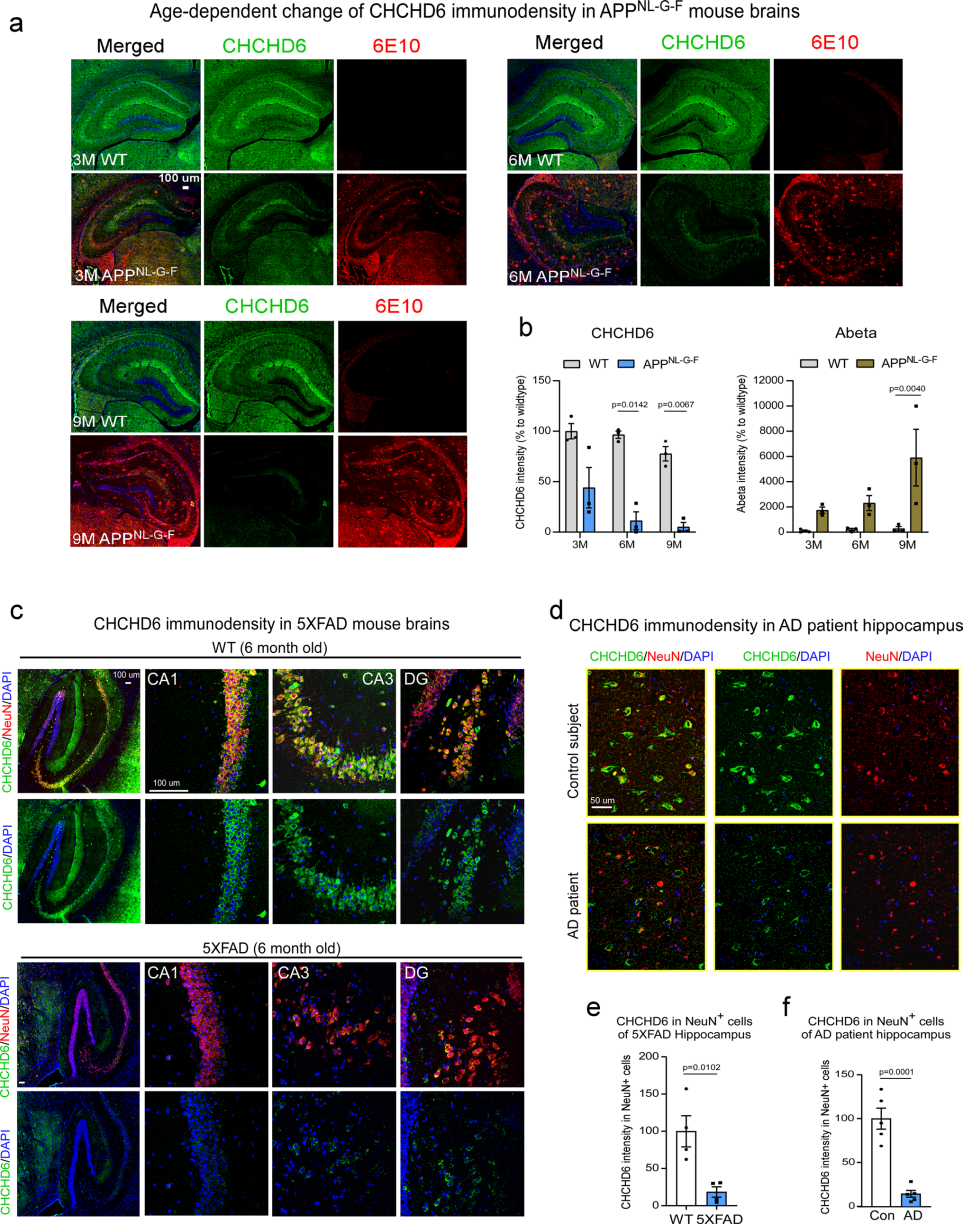
CHCHD6 decreases in neurons of AD mice and AD patient brains. **a** Brain sections from WT and APP^NL−G−F^ mice at the ages of 3, 6, and 9 months were stained with anti-CHCHD6 and anti-6E10 antibodies. The CHCHD6 and 6E10 immunodensities (*n* = 3 mice/group) were quantified and shown in (**b**). **c** Brain sections from 6-month-old WT and 5XFAD mice were stained with anti-CHCHD6 and anti-NeuN antibodies. The CHCHD6 immunodensity in NeuN^+^ cells (*n* = 4 mice/group) was quantified and shown in (**e**). **d** Postmortem hippocampus sections from control subjects and AD patients were stained with anti-CHCHD6 and anti-NeuN antibodies (*n* = 5 individuals/group). The intensity of CHCHD6 in NeuN^+^ cells was quantified and shown in (**f**). DAPI was used to label nuclei. All data are presented as mean ± SEM. The data in panel **b** were compared by two-way ANOVA with Tukey’s post hoc test, and the data in panels **e**, **f** were compared by the unpaired Student’s *t* test

We next visualized hippocampal CHCHD6 expression in AD mice and human AD brain tissue by immunofluorescence, and observed age-dependent decreases in APP^NL−G−F^ and 5XFAD mice, compared to age-matched WT mice (Fig. 2a and b, Supplementary Fig. 2a and b). Importantly, decreased CHCHD6 immunodensity was concomitant with increased accumulation of Aβ plaques (Fig. 2a and b, Supplementary Fig. 2a and b). CHCHD6 was mainly expressed in NeuN^+^ neurons, and its expression decreased even while the number of NeuN^+^ cells in the hippocampus remained the same between WT and AD mice (Fig. 2c and e). Similarly, CHCHD6 intensity was greatly decreased in NeuN^+^ neurons of human AD hippocampus and cortex, compared to normal subjects (Fig. 2d and f, Supplementary Fig. 2d). By contrast, CHCHD6 expression in Iba1^+^ microglia and GFAP^+^ astrocytes was substantially lower (Supplementary Fig. 2c), indicating neuronal enrichment of CHCHD6 expression.

### CHCHD6 and APP physically stabilize one another, and loss of CHCHD6 promotes aberrant APP processing

Exposure of neuroblastoma cells to toxic Aβ_1-42_, the APP cleavage product, did not alter protein or mRNA levels of CHCHD6, mitofilin, or CHCHD3 (Supplementary Fig. 3a and b). This indicated that a different aspect of amyloid biology must be affecting the integrity of the MICOS. To further investigate the molecular role of CHCHD6 in AD, we conducted a computational analysis to identify genes, pathways, phenotypes and diseases associated with CHCHD6 at genetic, functional, phenotypic and disease levels (Supplementary Fig. 3c). We prioritized biomedical entities based on the context-sensitive network-based ranking algorithm that we previously developed [11, 78], which revealed a number of MICOS genes significantly associated with CHCHD6. This validated the effectiveness of this virtual screening approach. Notably, APP emerged as the top candidate predicted to be associated with CHCHD6, ranking 0.003% among 30,049 human genes screened (Supplementary Fig. 3d). The top ranked predicted pathways for CHCHD6 were “protein metabolism,” “mitochondrial protein import,” “immune response,” “metabolism of lipids and lipoproteins,” and “Alzheimer’s disease” (Supplementary Fig. 3e). Moreover, CHCHD6 was also closely associated with human AD-related phenotypes among 49,639 human diseases and phenotypes screened (Supplementary Fig. 3e). Thus, the data strongly suggested that CHCHD6 might biologically intersect with APP, and thus potentially be involved in the pathophysiology of AD.

**Fig. 3:**
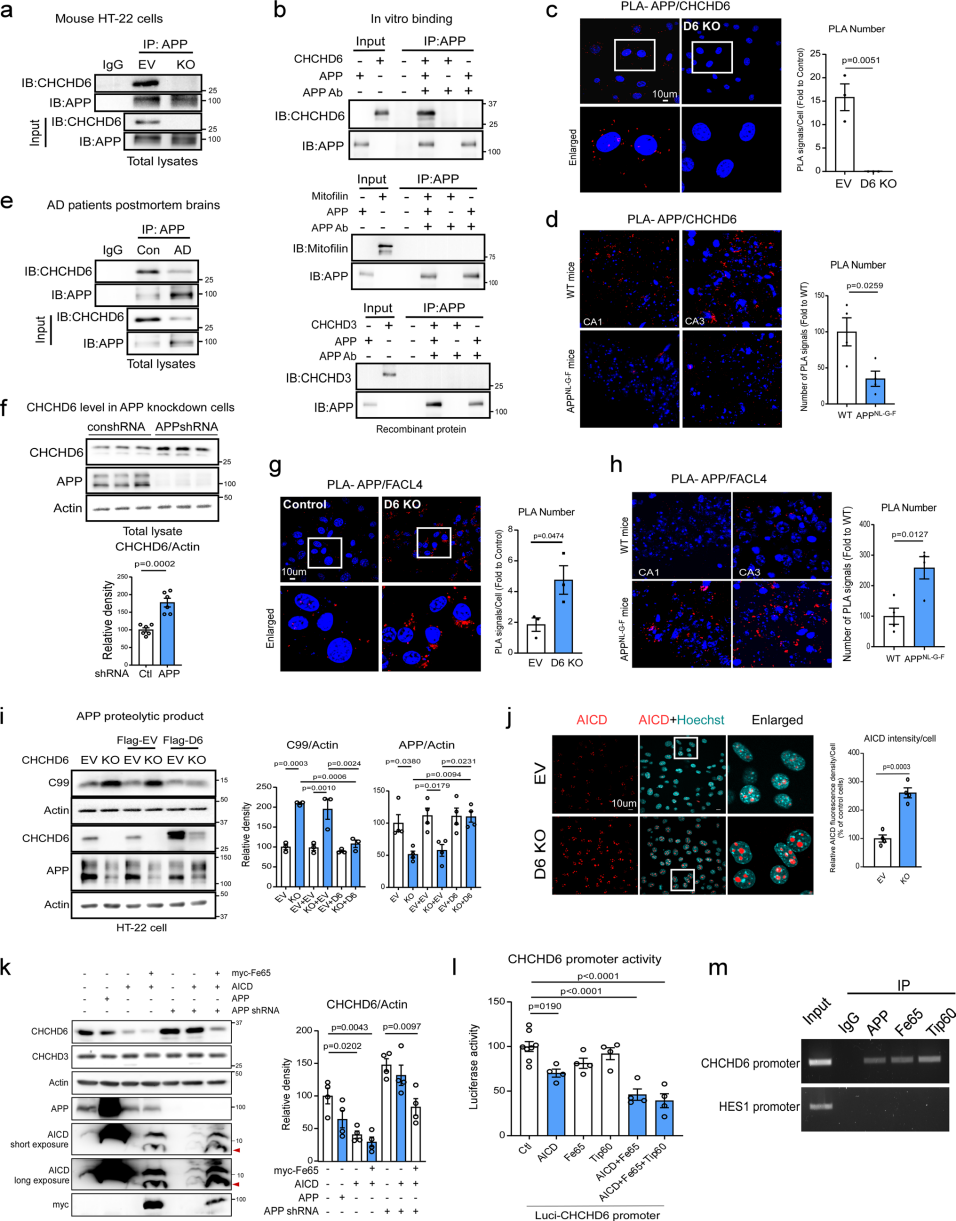
CHCHD6 and APP are interdependent to regulate each other. **a**. The total protein lysates of control and CHCHD6 KO HT-22 cells were subjected to immunoprecipitation (IP) with anti-APP antibody, followed by WB. Shown blots are representative of three independent experiments. **b** APP recombinant protein (500 ng) was incubated with either CHCHD6, Mitofilin or CHCHD3 recombinant proteins (500 ng, each). Immunoprecipitates with anti-APP antibodies was analyzed by immunoblotting with the indicated antibodies. Data are representative of 2 independent experiments. Control and CHCHD6 KO HT-22 cells were stained with anti-APP and anti-CHCHD6 antibodies (**c**) or anti-APP and anti-FACL4 antibodies (**g**), and subjected to in situ Duolink proximity ligation assay (PLA) analysis. Histogram shows the number of PLA-positive puncta (red). At least 47 cells/group (for APP and CHCHD6) and 250 cells/group (for APP and FACL4) were analyzed. The data were from 3 independent repeats. Brain sections from 6-month-old WT and APP^NL−G−F^ mice (*n* = 4 mice/group) were stained with anti-APP and anti-CHCHD6 antibodies (**d**) or anti-APP and anti-FACL4 antibodies (**h**), and subjected to PLA analysis. Histogram: the number of PLA-positive puncta (red) was quantified from at least four separate fields of each sample. **e** The total protein lysates of hippocampus of control subjects and AD patients were subjected to immunoprecipitation (IP) with anti-APP antibody, followed by WB. Shown blots are representative of three independent experiments. **f** HEK293 cells were infected with control or APP shRNA lentivirus. Total protein lysates were analyzed by WB with indicated antibodies. Histograms: relative density of CHCHD6 to actin. *n* = 6 independent experiments. **i** Control and CHCHD6 KO HT-22 cells were transfected with Flag empty vector (Flag-EV), or Flag-tagged CHCHD6 (D6-Flag) plasmids for 2 days. The total protein lysates were subjected to WB with the indicated antibodies. Histogram: the relative density of C99 and APP to actin. *n* = 3 independent experiments for C99 and *n* = 4 independent experiments for APP. **j** Control and CHCHD6 KO HT-22 cells were stained with anti-AICD antibody and Hoechst probe. The intensity of AICD was quantified and shown in the histogram. At least 300 cells/group were analyzed, and the data were from 4 independent experiments. **k** HEK293 cells were infected with control or APP shRNA lentivirus, and then transfected with the indicated plasmids for 72 h. The total protein lysates were subjected to WB. Red arrow: AICD. Histogram: the relative density of CHCHD6 to actin. *n* = 4 independent experiments. **l** HEK293 cells were transfected with CHCHD6 promoter-luc along or together with AICD, and/or Fe65 and/or Tip60 for 36 h. Cells were lysed, and the luciferase activity was measured. *n* = 8 for control and *n* = 4 for other groups. **m** HEK293 cells were transfected with GFP-labeled AICD, myc-Fe65 and Tip60-flag. CHIP analysis on CHCHD6 promoter was carried out with indicated antibodies. Hes1 gene ChIP is reported as a control. *n* = 2 independent experiments. All the data are mean ± SEM. The data in panel **c**, **d**, **f**–**h**, **j** were compared by the unpaired Student’s *t* test**,** and the data in panels **i**, **k** and **l** were compared by one-way ANOVA with Tukey’s post hoc test

Since APP has been localized to mitochondria, we next examined whether CHCHD6 and APP might directly interact. Indeed, immunoprecipitation of APP from mouse hippocampal HT-22 cells, which exhibit a large and tubular mitochondrial network, revealed physical interaction between CHCHD6 and APP, which was confirmed by CHCHD6 CRISPR-cas9 knockout (KO) (Fig. 3a, Supplementary Fig. 3f). In vitro binding assay of recombinant proteins also demonstrated a direct and selective interaction between CHCHD6 and APP, as there was no binding observed between APP and either mitofillin or CHCHD3 (Fig. 3b). Binding of APP and CHCHD6 was further established by in situ proximity ligation assay (PLA), with PLA-positive puncta observed in control cells but not CHCHD6 KO cells (Fig. 3c). PLA-positive puncta between anti-APP and anti-CHCHD6 antibodies were also observed in WT, APP^NL−G−F^ KI, and 5XFAD mouse hippocampus, with significantly reduced interaction in AD mice (Fig. 3d, Supplementary Fig. 3g and h) likely due to the reduced levels of CHCHD6. We further demonstrated a robust direct interaction between CHCHD6 and APP in postmortem human hippocampus of normal subjects, with diminished interaction in human AD hippocampus (Fig. 3e), similar to the animal models. Consistent with our earlier observation that overexpression of WT or mutant APP caused decreased transcription of CHCHD6 (Fig. 1), we also observed that APP downregulation by shRNAs significantly increased the level of CHCHD6 (Fig. 3f). This indicated an inverse correlation between CHCHD6 and APP levels. Interestingly, we found that decreased CHCHD6 enhanced APP accumulation on MAMs, as evidenced by more positive PLA fluorescence puncta between APP and the MAM marker fatty acid-CoA ligase 4 (FACL4) in CHCHD6 KO cells compared to WT (Fig. 3g). Western blot of mitochondrial fractionations confirmed enhanced APP and C99 levels on the MAM of CHCHD6 KO cells (Supplementary Fig. 4a). There was also an increased number of PLA-positive puncta between APP and FACL4 in the hippocampus of APP^NL−G−F^ KI and 5XFAD mice compared to WT (Fig. 3h, Supplementary Fig. 4b and c). MAMs are intracellular sites of APP processing [55], and accumulation of the C99 cleavage product of APP (an amylogenic precursor of Aβ) at MAMs impairs mitochondrial bioenergetics, disrupts cellular lipid homeostasis, and disrupts membrane lipid composition [37, 45]. Indeed, in CHCHD6 KO cells we observed significant decrease in total APP protein level and enhancement of the C99 fragment (Fig. 3i). By contrast, re-expression of CHCHD6 restored APP level and reduced C99 level (Fig. 3i). Taken together, these results demonstrate that CHCHD6 and APP interact and stabilize one another, and that loss of CHCHD6 promotes APP accumulation on the MAMs for processing.

### The APP processing product AICD binds CHCHD6 promoter and inhibits CHCHD6 transcription

In addition to C99, another cleavage product of APP processing is AICD [28], which regulates gene transcription through direct binding of AICD–Fe65–Tip60 complex to gene promoters [8, 33, 39, 74]. Thus, we wondered whether AICD might be involved in the transcriptional repression of CHCHD6 that we observed to be associated with rising levels of APP. After APP proteolysis, AICD is translocated to the nucleus by binding to adaptor Fe65 and regulates gene transcription [49, 65]. Consistent with previous findings [20, 59], AICD immunoreactivity recognized by an AICD-specific antibody was mainly observed in the nucleus. Notably, knockout of CHCHD6 in HT-22 cells greatly enhanced the intensity of AICD in the nucleus (Fig. 3j). The results further support our hypothesis that CHCHD6 loss promotes APP processing, which also leads to the accumulation of AICD. Next, we overexpressed APP, GFP-labeled AICD, and/or myc-Fe65 (the adaptor protein that stabilizes AICD and potentiates its nuclear translocation [49]) in HEK293 cells in the presence or absence of APP shRNA (Fig. 3k). While CHCHD6 protein level was consistently decreased by transient APP overexpression, even greater reduction was achieved by expression of AICD alone, or by co-expression of AICD and Fe65 (Fig. 3k). Co-expression of AICD and Fe65 also suppressed elevation of CHCHD6 protein that was otherwise induced by APP knockdown (Fig. 3k).

Having shown that levels of AICD and Fe65 inversely correlated with levels of CHCHD6, we further examined whether AICD and Fe65 could directly bind the CHCHD6 promoter. The histone acetyltransferase Tip60 forms a complex with the cytoplasmic tail of APP and the nuclear adaptor protein Fe65, enhancing the binding capacity of the complex to target gene promoters [6]. We therefore overexpressed AICD and Fe65 together with Tip60 in HEK293 cells. A fragment at the 5’ region of the CHCHD6 gene was subcloned into a luciferase reporter vector pGL3, and promoter activity was measured after transfection of AICD, Fe65, or both, in the presence or absence of Tip60. Notably, co-expression of AICD and Fe65 significantly reduced the ability of the CHCHD6 promoter to drive luciferase expression (Fig. 3l). We then performed chromatin immunoprecipitation (ChIP) assay in cells co-expressing AICD, Fe65 and Tip60. After immunoprecipitation with antibodies against the APP C-terminal region, Fe65 or Tip60, the collected DNAs were used as templates for PCR to amplify the CHCHD6 promoter region. DNA fragments immunoprecipitated by all three antibodies, but not by normal rabbit IgG, were found to contain the CHCHD6 promoter (Fig. 3m). By contrast, immunoprecipitated DNA fragments did not contain the promoter region of HES1, a transcriptional factor of the basic helix-loop-helix family (Fig. 3m), indicating a selectivity for the CHCHD6 promoter. These results suggest that the AICD/Fe65/Tip60 complex binds directly to the CHCHD6 promoter to suppress its activity, and support our observation that APP, presenting in either highly expressed WT or mutant form, mediates transcriptional inhibition of CHCHD6 (Fig. 1, Supplementary Fig. 1).

### CHCHD6 deficiency induces mitochondrial damage and cell death

**Fig. 4:**
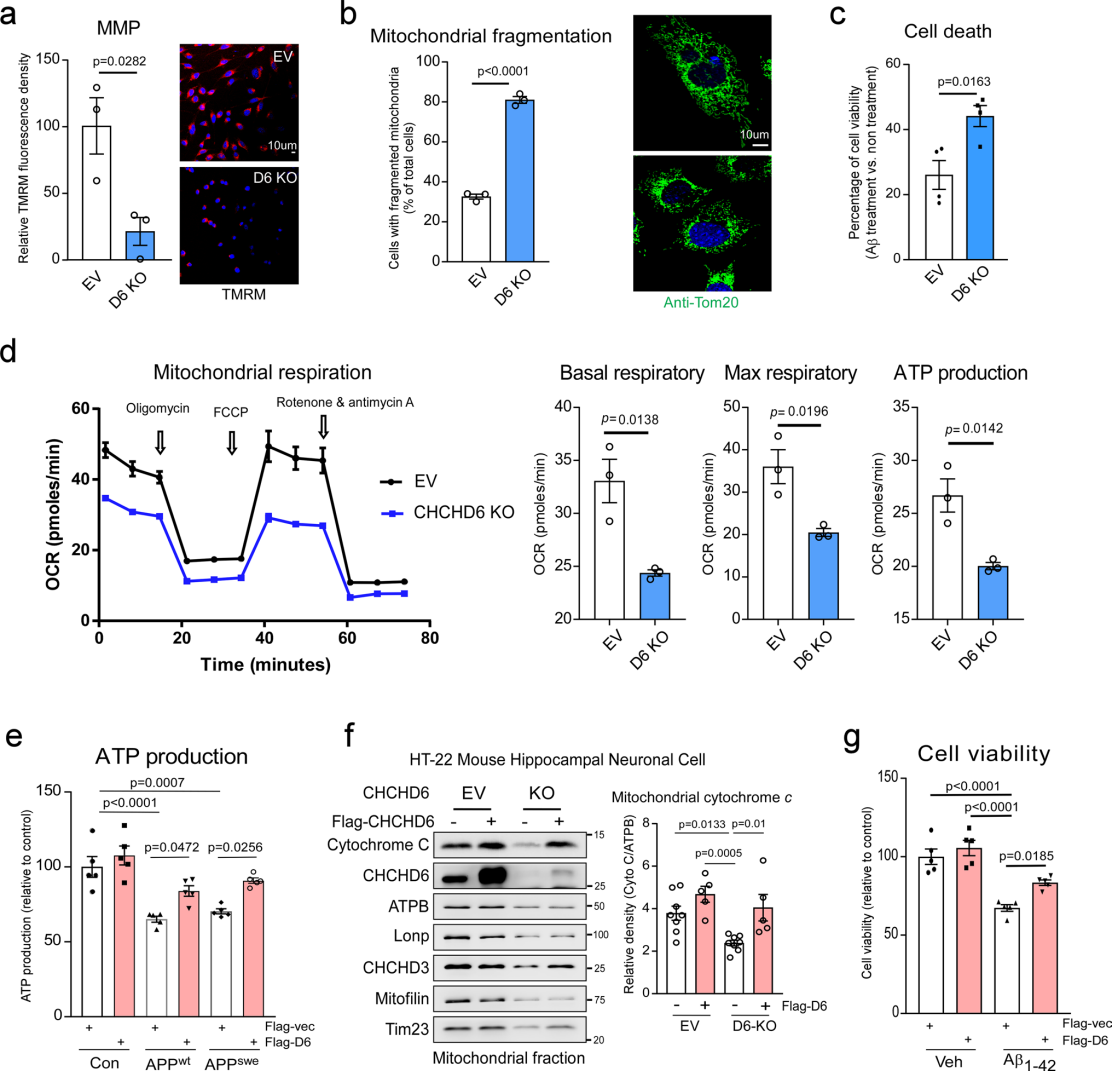
CHCHD6 deficiency induces mitochondrial impairment and cell damage. **a** Control and CHCHD6 KO HT-22 cells were stained with tetramethylrhodamine (TMRM) fluorescence dye. TMRM relative fluorescence density indicates the extent of mitochondrial membrane potential (MMP). Histogram: the relative fluorescence density of TMRM. At least 90 cells per group were analyzed, and the data were from 3 independent experiments. **b** Control and CHCHD6 KO HT-22 cells were stained with anti-Tom20 antibody (a mitochondrial marker, green) and Hoechst stain (nuclei, blue). Mitochondrial morphology was visualized using a 60X oil lens. The percentage of the cells with dot- or short bar-like fragmented mitochondria relative to the total number of cells was calculated and shown in the histogram (left). At least 50 cells per group were counted, and the data were from 3 independent experiments. Right: representative images. **c** Control and CHCHD6 KO HT-22 cells were treated with oligomeric Aβ_1–42_ peptides (5 μM) for 48 h and subjected to MTT assay. *n* = 4 independent experiments. **d** Mitochondrial respiratory activity of control and CHCHD6 KO HT-22 cells were measured using a Seahorse XFP analyzer and a Mito stress kit. OCR oxygen consumption rate. Basal respiration rate, maximal respiration rate, and ATP production are shown. *n* = 3 independent experiments. **e** Stable APP Neuro2a cells were transfected with Flag-EV or D6-Flag plasmids for 2 days, and then subjected to 16 h serum starvation. Cell death was measurement by MTT assay. *n* = 5 independent experiments. **f** Control and CHCHD6 KO HT-22 cells were transfected with Flag-EV or D6-Flag plasmids for 2 days. Protein lysates of mitochondria fraction were analyzed by WB with the indicated antibodies. Histograms: relative density of cytochrome C to ATPB. *n* = 8 for EV and KO control groups; *n* = 5 for D6-flag overexpressed groups. **g** HT-22 cells were transfected with Flag-EV or D6-Flag plasmids for 24 h and then treated with oligomeric Aβ_1–42_ peptides (5 μM) for 48 h. Cell death was measurement by MTT assay. *n* = 5 independent experiments. The data are presented as mean ± SEM. The data in panel **a**–**d** were compared by the unpaired Student’s *t* test, and the data in panels **e**–**g** were compared by one-way ANOVA with Tukey’s post hoc test

Next, we assessed the impact of CHCHD6 deficiency on mitochondrial integrity and neuronal survival. CHCHD6 KO in HT-22 cells led to mitochondrial depolarization, manifesting as a significant decline of mitochondrial membrane potential (MMP) (Fig. 4a). Downregulation of CHCHD6 increased the percentage of cells with Tom20^+^ dot or short bar-like mitochondria indicating mitochondrial fragmentation (Fig. 4b). CHCHD6 KO in cells also caused the accumulation of damaged mitochondria, as demonstrated by an increased number of LC3B^+^ puncta, elevated LC3II and p62 mitochondrial levels and decreased mitochondrial proteins from different sub-compartments (Supplementary Fig. 4d and e). These findings suggest the possibility of mitophagy impairment. In addition, CHCHD6 KO enhanced the sensitivity to Aβ-induced cell death (Fig. 4c). Consistent with previous studies [2, 16], deficiency in CHCHD6 suppressed mitochondrial respiratory activity, as manifested by reduced mitochondrial basal oxygen consumption rate, maximal oxygen consumption rate, and decreased ATP production in intact cells (Fig. 4d). Decreased ATP production was also observed in stable APP^wt^ and APP^swe^-overexpressing Neuro2a cells (Fig. 4e), in which the protein levels of CHCHD6 were low (Fig. 1b). Lower levels of mitochondrial cytochrome *c* were also detected in CHCHD6 KO HT-22 cells (Fig. 4f), consistent with the previous observation that loss of MICOS results in release of cytochrome *c* from mitochondria [18]. Compensation for the loss of CHCHD6 by overexpressing CHCHD6-Flag construct restored ATP production in stable APP Neuro2a cells (Fig. 4e), reduced cytochrome *c* release induced by CHCHD6 KO in HT-22 mouse hippocampal neurons (Fig. 4f), and attenuated Aβ-induced cell death (Fig. 4g). Taken together, these results demonstrate that CHCHD6 is required for maintenance of mitochondrial quality control and bioenergetics, and that loss of CHCHD6 perturbs cristae integrity leading to bioenergetic defects and cell death.

### CHCHD6 deficiency induces neuronal cholesterol accumulation in AD models

**Fig. 5:**
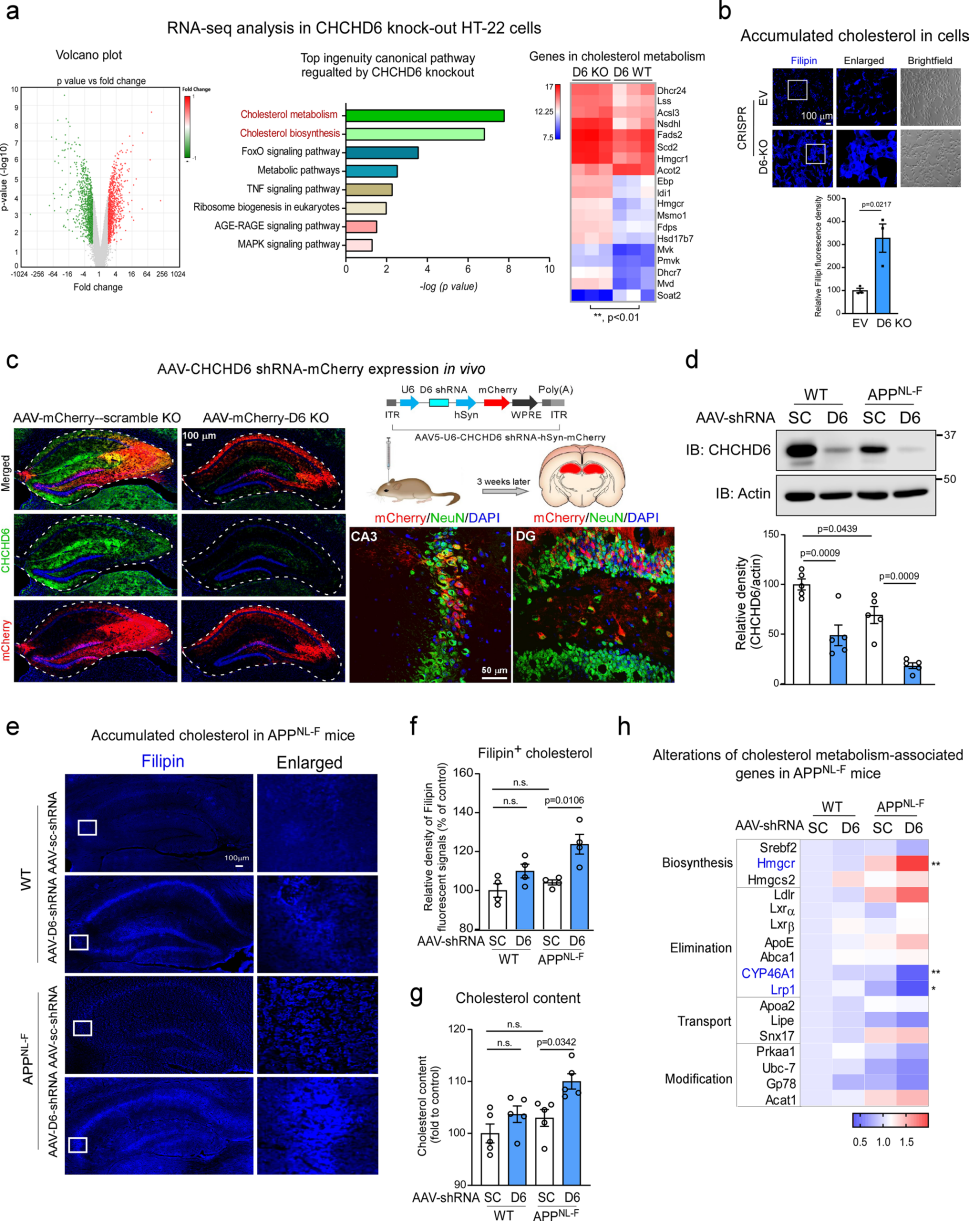
CHCHD6 deficiency induces neuronal cholesterol accumulation in AD models. **a** Control and CHCHD6 KO HT-22 cells (*n* = 3) was subjected to whole transcriptome RNA-Seq analysis. Left: RNA transcripts changed in CHCHD6 KO cells. Middle: KEGG database analysis on RNA transcripts that are twofold downregulated or upregulated relative to control cells. Right: Heat map: genes involved in the “Pikuleva metabolism”. **p < 0.01 (unpaired Student’s *t* test). **b** Control and CHCHD6 KO HT-22 cells were stained with the filipin probe. Histogram: the immunodensity of filipin^+^ cholesterol was quantified from ten separate fields per sample. *n* = 3 independent experiments. **c** AAV5-U6-CHCHD6 shRNA-hSyn-mCherry and AAV5-U6-Scramble shRNA-hSyn-mCherry were stereotaxically injected into bilateral hippocampus of WT or APP^NL−F^ mice at the age of 6 months. Image: AAV induced expression of mCherry in NeuN^+^ neurons in the hippocampus of WT or APP^NL−F^ mice 3 weeks after injection. Mice were sacrificed 6 months after injection of AAV-Scramble shRNA or AAV-CHCHD6 shRNA. **d** Total brain lysates were harvested from the hippocampus of 12-month-old mice at the indicated groups. CHCHD6 knockdown efficiency was assessed by WB. Histogram: the relative density of CHCHD6 to actin. *n* = 5 mice/group. **e** Brain sections from 12-month-old AAV-Scramble shRNA or AAV-CHCHD6 shRNA-injected WT or APP^NL−F^ mice were stained with the filipin probe. The immunodensity of filipin^+^ cholesterol was quantified and shown in **f** (*n* = 4 mice/group). **g** The total cholesterol content was measured in the hippocampus from 12-month-old AAV-Scramble shRNA or AAV-CHCHD6 shRNA-injected WT or APP^NL−F^ mice using ELISA kit (*n* = 5 mice/group). **h** mRNAs were extracted from 12-month-old AAV-Scramble shRNA or AAV-CHCHD6 shRNA-injected WT or APP^NL−F^ mice. Genes involved in cholesterol metabolism were analyzed by qPCR (*n* = 5 mice/group). Heat map: the mean of the genes analyzed. *p < 0.05; **p < 0.01 (AAV-Scramble shRNA-injected APP^NL−F^ mice vs. AAV-CHCHD6 shRNA-injected APP^NL−F^ mice. The data are presented as mean ± SEM. The data in panel **d**, **f**–**h** were compared by one-way ANOVA with Tukey’s post hoc test

To further evaluate the impact of CHCHD6 deficiency on AD-associated neuronal damage, we conducted whole transcriptome RNA-Seq analysis of CHCHD6 KO HT-22 mouse hippocampal neurons. Among the 5336 RNA transcripts identified in control HT-22 cells, we focused on RNAs that were altered in CHCHD6 KO HT-22 cells (i.e., > twofold downregulation or upregulation relative to control cells; Fig. 5a). A total of 1827 RNAs that met these criteria (Fig. 5a, green and red dots) were then used for pathway enrichment analysis. Graphical comparison of the KEGG analysis showed that cholesterol metabolism and cholesterol biosynthesis ranked as the top two RNA enrichment pathways, and genes involved in cholesterol metabolism were dramatically altered in CHCHD6 KO cells (Fig. 5a). In parallel, we stained control and CHCHD6 KO HT-22 cells with filipin, a fluorescence probe used to monitor cholesterol deposition. We observed significantly more filipin-bound cholesterol in CHCHD6 KO HT-22 cells than in control cells (Fig. 5b). Accumulation of cholesterol is synaptotoxic in AD [53], and our results suggest that loss of CHCHD6 may promote cholesterol accumulation in neurons by regulating expression of cholesterol metabolism genes.

To test the effects of CHCHD6 loss on accumulation of neuronal cholesterol level in vivo, we used shRNA against CHCHD6 expressed by adeno-associated viral (AAV) vector to specifically downregulate CHCHD6 in APP^NL−F^ mice and age-matched WT littermates (Supplementary Fig. 5a). A potent and specific CHCHD6 shRNA was inserted in the AAV5 vector under control of the U6 promoter co-expressing the fluorophore mCherry, itself under the control of the human synapsin (hSyn) promoter. This allowed selective expression restricted to neurons (Fig. 5c). We employed the same vector expressing a scrambled shRNA as a control, and stereotaxically injected either AAV5-scrambled shRNA or AAV5-CHCHD6 shRNA into bilateral hippocampus of WT or APP^NL−F^ mice at 6 months of age. At this age, APP^NL−F^ mice do not exhibit AD-associated neuropathology or behavioral impairment. Six months after stereotaxic injection, we observed that mCherry-labeled AAV containing CHCHD6 shRNA was successfully delivered (Fig. 5c). Compared to the mice injected with AAV5-scrambled shRNA, western blot analysis showed a strong reduction of CHCHD6 in hippocampal protein extracts of AAV5-CHCHD6 shRNA-injected mice (Fig. 5d). Downregulation of CHCHD6 in neurons was also demonstrated by significantly decreased CHCHD6 intensity in NeuN^+^ cells in the hippocampus of mice, while no significant change in the number of NeuN^+^ cells among experimental groups was observed (Supplementary Fig. 5b). This reduced level of CHCHD6 is not due to mitochondria loss, given the presence of comparable levels of the mitochondrial proteins voltage-dependent anion channel (VDAC: an outer membrane proteins), translocase of the inner mitochondrial membrane (Tim23: an inner membrane protein) and lon protease-like protein (LonP: a matrix protein) across all groups (Supplementary Fig. 5c). Moreover, AAV-mediated knockdown was specific for CHCHD6 and had no effects on other subunits of MICOS complex, such as mitofilin and CHCHD3 (Supplementary Fig. 5d).

Notably, injection of AAV5-CHCHD6 shRNA caused elevated levels of filipin^+^ cholesterol in the hippocampus of both WT and APP^NL−F^ mice 6 months later, with significantly more cholesterol accumulation observed in APP^NL−F^ mice (Fig. 5e and f). ELISA analysis confirmed that the increased accumulation of cholesterol was caused by CHCHD6 reduction, showing increased total cholesterol content in the hippocampus of APP^NL−F^ mice injected with AAV5-CHCHD6 shRNA (Fig. 5g). Considering that cholesterol metabolism was disrupted in CHCHD6 KO HT-22 neuronal cells (Fig. 5a), we next examined which pathway of cholesterol metabolism was most influenced by reduced CHCHD6 by analyzing genes implicated in cholesterol biosynthesis, elimination, transport and modification. In both CHCHD6 KO HT-22 cells and APP^NL−F^ KI mice injected with AAV5-CHCHD6 shRNA, *hmgcr*, *cyp46a1* and *lrp1* showed consistent alterations (Fig. 5h, Supplementary Fig. 5e). *Hmgcr* encodes HMG-CoA reductase (HMGCR), which converts HMG-CoA to mevalonic acid as the rate-limiting enzyme in cholesterol biosynthesis [35]. *Cyp46A1* encodes cytochrome P450 family 46 subfamily A member 1 (CYP46A1), which is a neuronal-specific and rate-limiting enzyme that catalyzes 24-hydroxylation of cholesterol, the main process for cholesterol elimination from the brain [17]. Notably, augmentation of CYP46A1 has been shown to be beneficial in an animal model of AD [22, 47]. In APP^NL−F^ mouse hippocampus injected with AAV5-CHCHD6 shRNA, the mRNA level of *hmgcr* significantly increased, whereas the mRNA level of *cyp46a1* markedly decreased, compared to mice injected with AAV5-scrambled shRNA (Fig. 5h). Thus, CHCHD6 downregulation may promote neuronal cholesterol accumulation in APP^NL−F^ AD mice by enhancing cholesterol biosynthesis and suppressing cholesterol elimination. Lastly, *lrp1* encodes LDL receptor related protein 1 (LRP1), an endocytic receptor in neurons that mediates uptake of cholesterol-ApoE-containing lipoproteins from astrocytes [24]. Transcription of LRP1 is also suppressed by AICD through direct binding of AICD–Fe65–Tip60 complex to its promoter [33]. Given that CHCHD6 deficiency promotes APP processing (Fig. 3i) and thereby generates more AICD (Fig. 3j), decreased mRNA level of *lrp1* upon knocking down CHCHD6 is not surprising (Fig. 5h, Supplementary Fig. 5e).

### Decreased CHCHD6 accelerates cognitive deficits and AD pathology in APPNL^−F^ KI mice

**Fig. 6:**
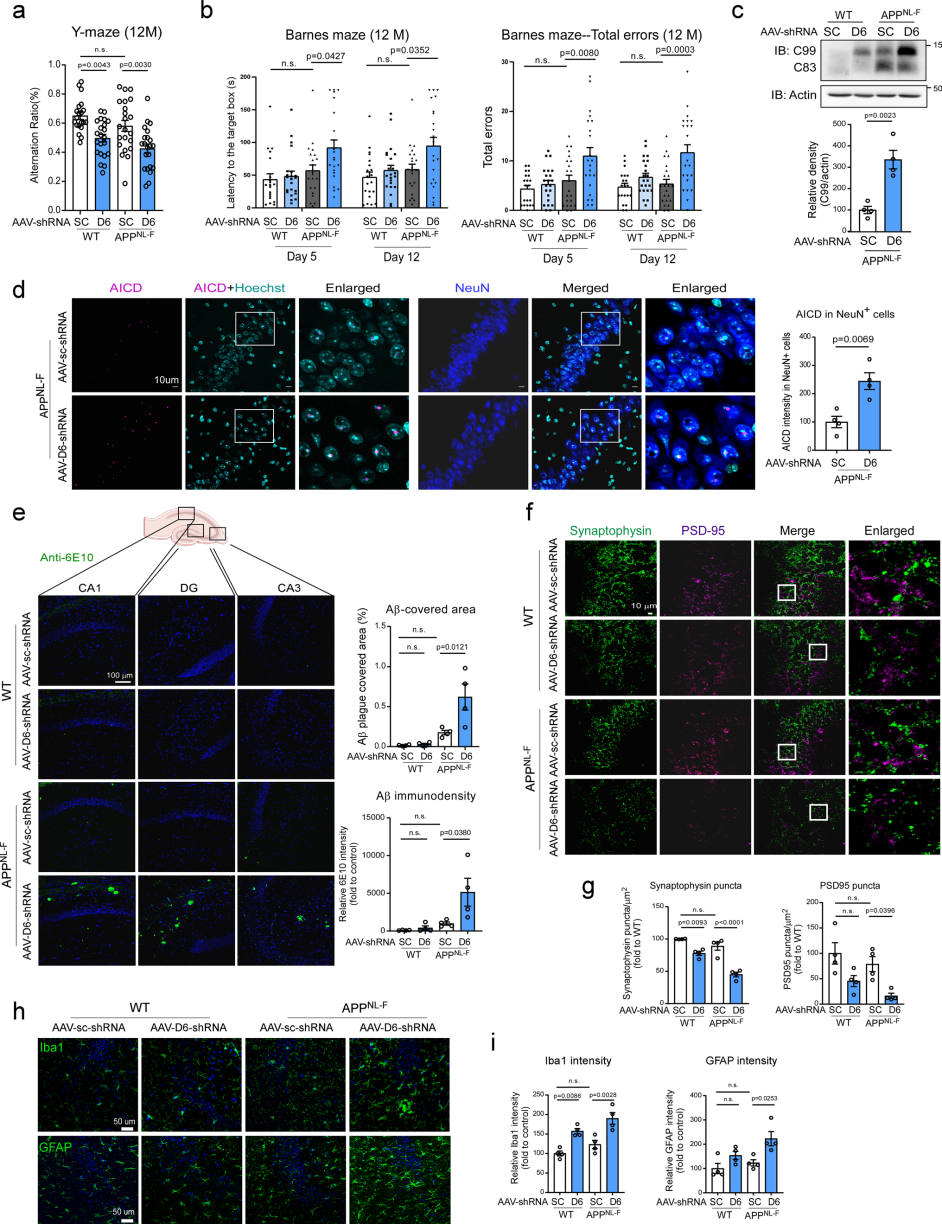
Viral vector-mediated downregulation of CHCHD6 accelerates cognitive deficits and AD pathology in APP^NL−F^ KI mice. **a** The Y-maze test was performed with 12-month-old mice (WT/Scramble shRNA: *n* = 20 mice; *n* = 22 mice/group for the WT/CHCHD6 shRNA, and APP^NL−F^/Scramble shRNA, and APP^NL−F^/CHCHD6 shRNA groups). **b** The Barnes maze test was performed with 12-month-old mice (WT/Scramble shRNA: *n* = 20 mice; *n* = 22 mice/group for the WT/CHCHD6 shRNA, and APP^NL−F^/Scramble shRNA, and APP^NL−F^/CHCHD6 shRNA groups). **c** Total brain lysates were harvested from the hippocampus of 12-month-old AAV-Scramble shRNA or AAV-CHCHD6 shRNA-injected WT or APP^NL−F^ mice. The level of C99 was analyzed by WB. Histogram: the relative density of C99 to actin (*n* = 4 mice/group). **d** Brain sections from 12-month-old mice were stained with anti-AICD and anti-NeuN. Histogram: the intensity of AICD in NeuN^+^ cells was quantified from four separate fields of each mouse (*n* = 4 mice/group). **e** Brain sections from 12-month-old AAV-Scramble shRNA or AAV-CHCHD6 shRNA-injected WT or APP^NL−F^ mice were stained with an anti-6E10 antibody. Histograms: the area covered by 6E10^+^ Aβ plaques and the immunodensity of 6E10 in hippocampus were quantified from four separate fields of each mouse (*n* = 4 mice/group). **f** Brain sections of mice with the indicated groups were stained with anti-PSD95 and anti-synaptophysin antibodies. The numbers of PSD95^+^ and synaptophysin^+^ puncta were quantified and shown in **g** (*n* = 4 mice/group). **h** Brain sections of mice with the indicated groups were stained with anti-Iba1 and anti-GFAP antibodies. The intensity of Iba1 and GFAP was quantified and shown in (**i)** (*n* = 4 mice/group). The data are presented as mean ± SEM. The data in panel **a**, **b**, **e**, **g**, **i** were compared by one-way ANOVA with Tukey’s post hoc test, and the data in panel **c** and **d** were compared by the unpaired Student’s *t* test

Next, we investigated whether downregulation of CHCHD6 affected AD-like neuropathology and behavioral deficits in the slowly developing APP^NL−F^ KI mice. At 12 months of age (6 months after AAV injection), WT and APP^NL−F^ mice injected with AAV-scrambled shRNA showed the same performance in the Y-maze test of cognition, confirming a lack of AD phenotype at this age [54]. With AAV5-CHCHD6 shRNA injection, however, we observed a decline in the spontaneous alteration ratio in the Y-maze test in both WT and APP^NL−F^ mice (Fig. 6a). The number of total arm entries during the Y-maze test among the experimental groups was not statistically different, suggesting that knockdown of CHCHD6 in WT and APP^NL−F^ mice did not influence sensorimotor capacities (Supplementary Fig. 6a). Furthermore, while no difference was found in the Barnes maze test of cognition between 12-month-old WT and APP^NL−F^ mice injected with scrambled shRNA, AAV5-CHCHD6 shRNA-injected APPNL^−F^ KI mice took a longer time and made more errors in finding the target escape area (Fig. 6b). There was no significant difference in body weight among groups (Supplementary Fig. 6b). Taken together, these data indicate that CHCHD6 deficiency in chronic APP^NL−F^ AD mice promotes impairment of spatial learning and long-term memory.

To examine whether pathological markers of AD were also affected by AAV-shRNA-mediated knockdown of CHCHD6, we first checked the level of APP processing products in protein extracts of hippocampus at 12 months of age. Western blot analysis revealed that AAV-CHCHD6 shRNA injection enhanced APP processing in both WT and APP^NL−F^ mice, showing elevated level of C99 fragment (Fig. 6c). There was a much higher level of C99 product in AAV-CHCHD6 shRNA-injected APP^NL−F^ mice than in mice injected with AAV-scrambled shRNA (Fig. 6c). These results were consistent with more APP accumulation on the MAM in AAV-CHCHD6 shRNA-injected APP^NL−F^ mouse brains (Supplementary Fig. 6c). Furthermore, injection of AAV-CHCHD6 shRNA elevated the AICD intensity in NeuN^+^ cells of APP^NL−F^ mice compared with injection of AAV-scrambled shRNA (Fig. 6d). We also stained brain sections of 12-month-old mice with anti-6E10 antibody to label amyloid aggregation. Though there was no statistical difference between WT and APP^NL−F^ mice, we observed a higher number of 6E10^+^ amyloid depositions and a larger area covered by amyloid plagues in the hippocampus of AAV-CHCHD6 shRNA-injected APP^NL−F^ mice, relative to APP^NL−F^ mice injected with scrambled shRNA (Fig. 6e). These data are consistent with our findings in HT-22 cells (Fig. 3i and j), supporting our conclusion that loss of CHCHD6 promotes APP processing, which then accelerates amyloid pathology.

As synapse loss strongly correlates with cognitive decline in AD [21], we stained mouse brain sections with antibodies against synaptophysin (a presynaptic marker) and post-synaptic density 95 (PSD95: a postsynaptic marker). We observed a marked decrease in the number of synaptophysin- and PSD95-immunoreactive puncta in the CA3 region of APP^NL−F^ mice after AAV-CHCHD6 shRNA injection, indicating decreased synaptic density (Fig. 6f and g). Western blot analysis confirmed the decline in synaptophysin and PSD95 protein levels in the hippocampus of AAV-CHCHD6 shRNA-injected APP^NL−F^ mice (Supplementary Fig. 6d). We next examined neuroinflammation, another pathological marker of AD. Immunofluorescence staining showed that the intensities of ionized calcium binding adaptor molecule 1 (Iba1: a marker of microglia) and glial fibrillary acidic protein (GFAP: a marker of astrocytes) significantly increased in the hippocampus of AAV-CHCHD6 shRNA-injected APP^NL−F^ mice (Fig. 6g and i), compared to the mice injected with scrambled shRNA control. This indicates enhanced AD-associated neuroinflammation by CHCHD6 downregulation. Collectively, these data demonstrate that downregulation of CHCHD6 accelerates neuropathology and cognitive deficits in AD mice.

### Compensation for loss of CHCHD6 reduces neuropathology and cognitive deficits in APPNL^−G−F^ KI mice

**Fig. 7:**
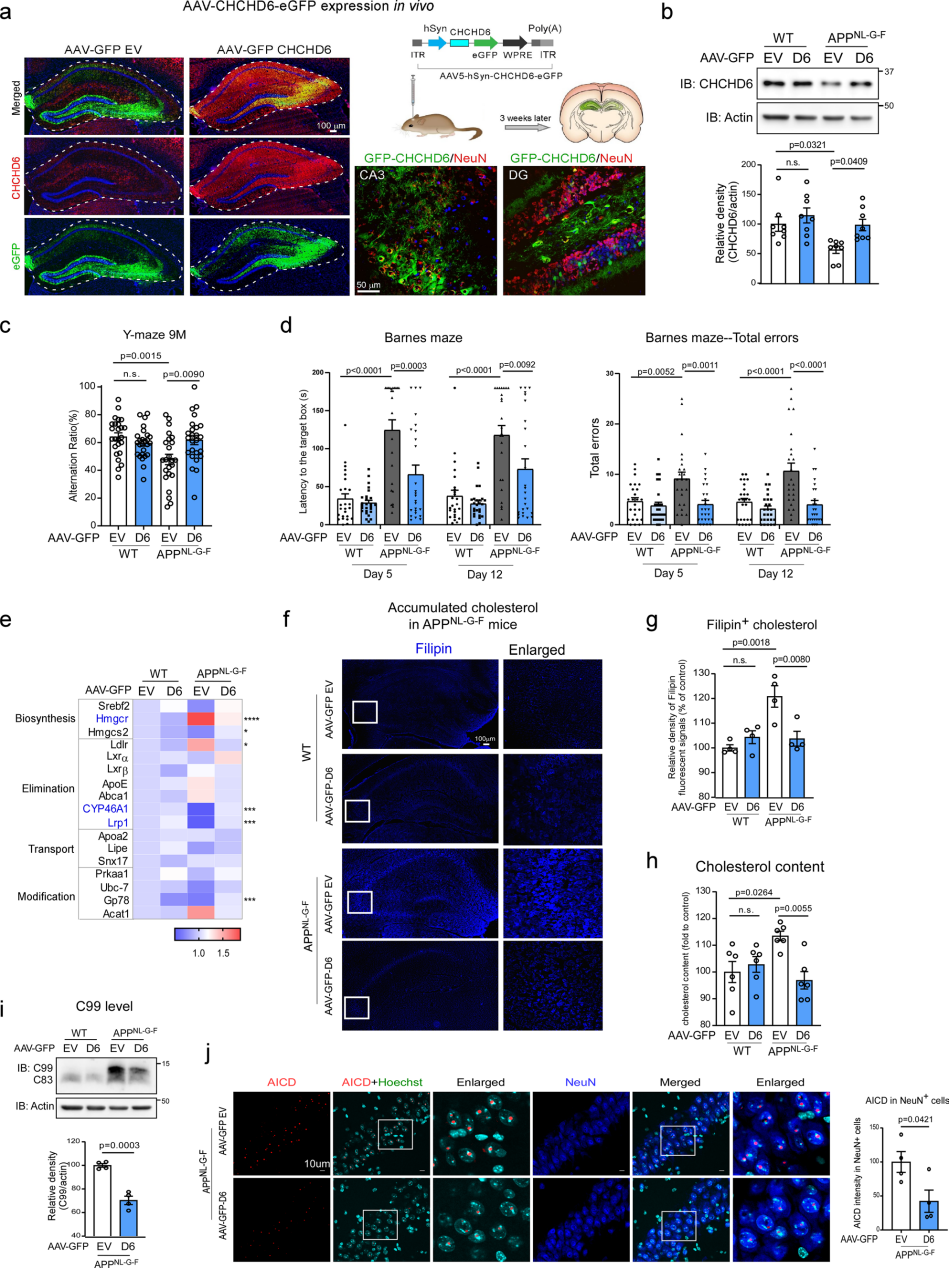
Compensation for the loss of CHCHD6 reduces neuropathology and cognitive deficits in APP^NL−G−F^ KI mice. **a** AAV5-hSyn-CHCHD6-eGFP and AAV5-hSyn-eGFP control were stereotaxically injected into bilateral hippocampus of 3-month-old WT or APP^NL−G−F^ mice. Image: AAV induced expression of eGFP in NeuN^+^ neurons in mouse hippocampus 3 weeks after injection. Mice were killed 6 months after AAV injection. **b** Total brain lysates were harvested from the hippocampus of 9-month-old mice. WB was performed with the indicated antibodies. Histogram: the relative density of endogenous CHCHD6 to actin (*n* = 5 mice/group). **c** The Y-maze test was performed with 9-month-old mice (WT/EV and APP^NL−G−F^/CHCHD6 groups: *n* = 25 mice; *n* = 26 mice for WT/CHCHD6; and *n* = 24 mice for APP^NL−G−F^/EV). **d** The Barnes maze test was carried out with 9-month-old mice (WT/EV and APP^NL−G−F^/CHCHD6 groups: *n* = 25 mice; *n* = 26 mice for WT/CHCHD6; and *n* = 24 mice for APP^NL−G−F^/EV). **e** mRNAs were extracted from 9-month-old mice at the indicated groups. Genes involved in cholesterol metabolism were analyzed by qPCR (*n* = 5 mice/group). Heat map: the mean of the genes analyzed. *p < 0.05; ***p < 0.001; ****p < 0.0001; (AAV-EV-injected APP^NL−G−F^ mice vs. AAV-CHCHD6-injected APP^NL−G−F^ mice). **f** Brain sections from 9-month-old mice at the indicated groups were stained with the filipin probe. The immunodensity of filipin^+^ cholesterol was quantified and shown in (**g**) (*n* = 4 mice/group). **h** The total cholesterol content was measured in the hippocampus from 9-month-old mice using ELISA kit (*n* = 6 mice/group). **i** Total brain lysates were harvested from the hippocampus of 9-month-old mice. The level of C99 was analyzed by WB. Histogram: the density of C99 relative to actin (*n* = 4 mice/group). **j** Brain sections from 9-month-old AAV-CHCHD6 or AAV-EV-injected APP^NL−G−F^ mice were stained with anti-AICD and anti-NeuN. The intensity of AICD in NeuN^+^ cells was quantified from four separate fields of each mouse (*n* = 4 mice/group). The data in panel **b**–**e**, **g**, **h** were compared by one-way ANOVA with Tukey’s post hoc test, and the data in panel **i**, **j** were compared by the unpaired Student’s *t* test

Lastly, we addressed the complementary question of whether compensating for CHCHD6 loss would attenuate AD-like neuropathology and cognitive deficits in APP^NL−F−G^ AD mice. CHCHD6 was inserted into an AAV vector under control of the hSyn promoter (AAV5-hSyn-CHCHD6-eGFP), to achieve selective expression of GFP-labeled CHCHD6 in neurons (Fig. 7a). AAV5-hSyn-eGFP was used as empty vector control. AAV5-hSyn-CHCHD6-eGFP, or AAV control construct, were stereotaxically injected in bilateral hippocampus of 3-month-old APP^NL−G−F^ mice and age-matched WT littermates (Supplementary Fig. 7a). Six months later, in contrast to low protein level of CHCHD6 in AAV5-eGFP-injected APP^NL−F−G^ mice, western blot analysis revealed significant upregulation of CHCHD6 protein level in hippocampal protein extracts of AAV5-eGFP-CHCHD6-injected APP^NL−F−G^ mice, to a similar level as WT mice injected with AAV5-eGFP control (Fig. 7b). AAV-mediated overexpression of CHCHD6 did not influence mitochondrial mass, as evidenced by comparable levels of proteins from different mitochondrial subcompartments (Supplementary Fig. 7b). Viral overexpression of CHCHD6 also did not alter protein levels of mitofilin and CHCHD3 (Supplementary Fig. 7c).

AAV5-eGFP control injected APP^NL−G−F^ mice displayed decreased short-term cognitive ability, as assessed by the Y-maze at 9 months of age. By contrast, age-matched APP^NL−G−F^ mice injected with AAV5-eGFP-CHCHD6 had an improved spontaneous alteration ratio in the Y-maze test, reaching levels similar to WT mice (Fig. 7c). Importantly, all groups displayed an equivalent number of total arm entries of the Y-maze test (Supplementary Fig. 7d). These results suggest that CHCHD6 neuronal overexpression improves spatial working memory of APP^NL−F−G^ AD mice. In addition, CHCHD6 overexpression also significantly improved the performance of APP^NL−G−F^ mice in the Barnes maze test at 9 months of age (Fig. 7d). AAV-CHCHD6-injected APP^NL−G−F^ mice took a shorter time and made fewer errors in finding the target escape area than mice injected with AAV-eGFP control. Expression of AAV-eGFP-CHCHD6 in WT mice did not alter animal behavior (Fig. 7c and d), and the body weight of mice among experimental groups was comparable (Supplementary Fig. 7e), indicating lack of toxicity of viral overexpression.

We also assessed whether compensation for CHCHD6 loss could attenuate AD-related perturbation of cholesterol metabolism. Consistent with the results in APP^NL−F^ mice, expression analysis of cholesterol-related genes revealed that *hmgcr*, *cyp46a1* and *lrp1* were significantly affected by AAV-mediated CHCHD6 overexpression. AAV5-eGFP-CHCHD6 injection abolished the elevated cholesterol synthesis gene *hmgcr* and suppressed cholesterol elimination gene *cyp46a1*, which were otherwise observed in AAV5-eGFP control injected APP^NL−G−F^ mice (Fig. 7e). A significant increase in filipin^+^ cholesterol in the hippocampus of APP^NL−G−F^ mice injected with AAV-eGFP control was observed at the age of 9 months, which was reduced by viral expression of AAV-eGFP-CHCHD6 starting from the age of 3 months (Fig. 7f and g). Furthermore, ELISA analysis showed that AAV-eGFP-CHCHD6 expression abolished increased cholesterol content in the hippocampus of APP^NL−G−F^ mice (Fig. 7h). AAV-eGFP-CHCHD6 had no effects on cholesterol content in WT mice (Fig. 7h).

**Fig. 8:**
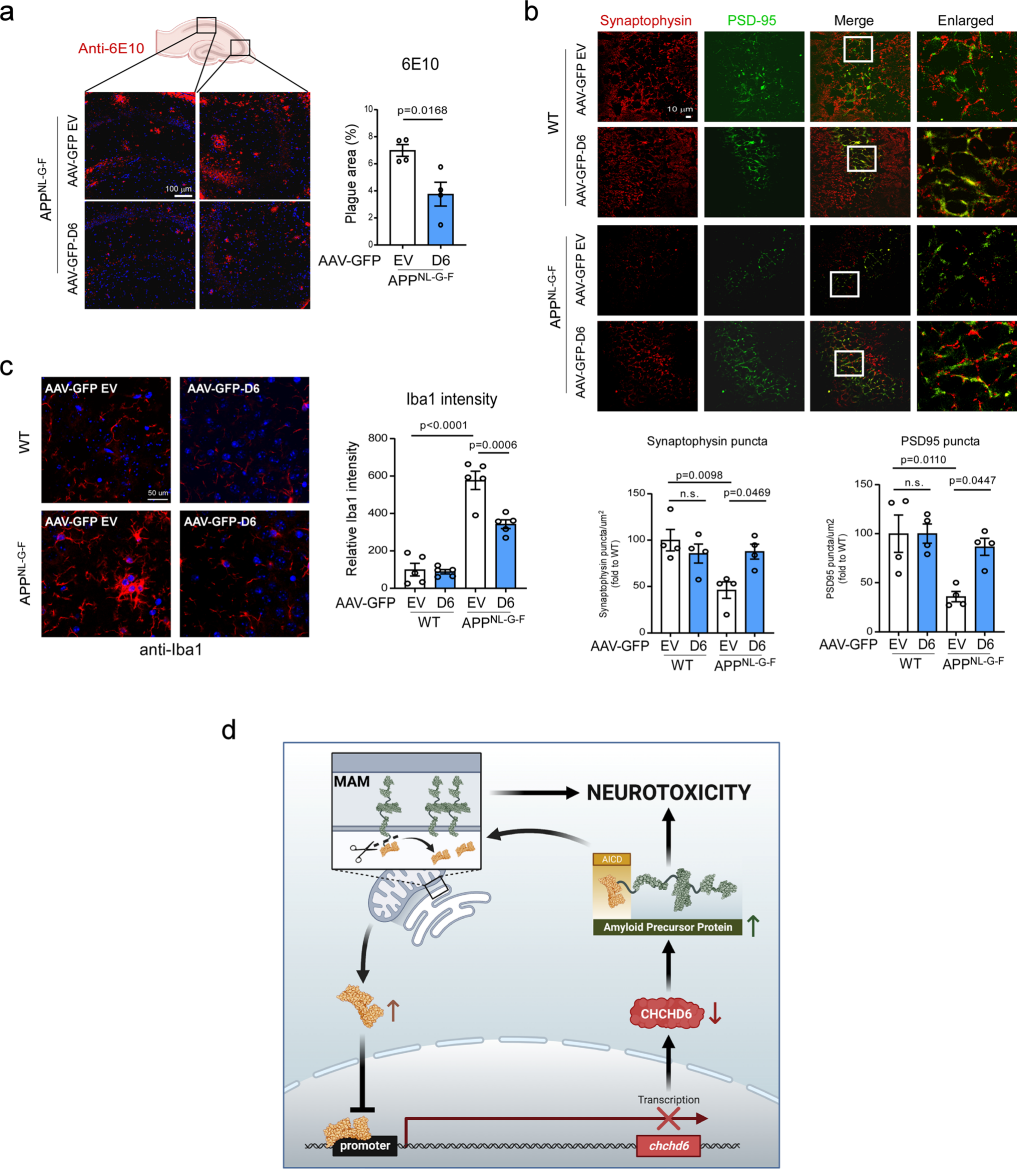
Compensation for the loss of CHCHD6 reduces amyloid accumulation, synaptic loss and gliosis in APP^NL−G−F^ KI mice. **a** Brain sections from 9-month-old AAV-CHCHD6 or AAV-EV-injected APP^NL−G−F^ mice were stained with anti-6E10 antibodies. Histograms: the area covered by 6E10^+^ Aβ plaques in hippocampus were quantified from four separate fields of each mouse (*n* = 4 mice/group). **b** Mouse brain sections were stained with anti-PSD95 and anti-synaptophysin antibodies. Histograms: the numbers of PSD95^+^ and synaptophysin^+^ puncta were quantified (*n* = 4 mice/group). **c** Mouse brain sections were stained with anti-Iba1 antibody. Histograms: the intensity of Iba1 was quantified (*n* = 5 mice/group). The data are presented as mean ± SEM. The data in panel a were compared by the unpaired Student’s *t* test, and the data in panel **b** and **c** were compared by one-way ANOVA with Tukey’s post hoc test. **d** A summarized scheme

Lastly, we also observed a significant increase in APP on the MAM and C99 in the hippocampus of 9-month-old APP^NL−F−G^ mice injected with AAV-eGFP control, compared to age-matched WT mice. By contrast, AAV-mediated CHCHD6 expression normalized APP accumulation on the MAM and lowered the level of C99 fragment in APP^NL−G−F^ mice, suggesting a suppression of APP processing (Supplementary Fig. 7f and i). CHCHD6 overexpression also reduced AICD intensity in NeuN^+^ cells and Aβ-covered area in the hippocampus of 9-month-old APP^NL−G−F^ mice, compared to mice injected with AAV-eGFP control (Figs. 7j and 8a). Furthermore, imaging analysis revealed significantly decreased synaptophysin and PSD95 in the hippocampus of AAV-eGFP control injected APP^NL−G−F^ mice at 9 months of age, with restoration to normal by AAV-mediated CHCHD6 overexpression (Fig. 8b). Western blot analysis also confirmed that AAV-mediated CHCHD6 overexpression in APP^NL−G−F^ mice restored hippocampal protein levels of synaptophysin and PSD95 (Supplementary Fig. 8). These findings are consistent with the improvement of cognitive activity observed in AAV-eGFP-CHCHD6-injected APP^NL−G−F^ mice (Fig. 7c and d). In addition, AAV5-eGFP-CHCHD6 overexpression significantly reduced Iba1 immunodensity in APP^NL−G−F^ mice, indicating an inhibition of neuroinflammation (Fig. 8c). Our results thus demonstrate that compensation for the loss of CHCHD6 in the hippocampus of rapidly developing APP^NL−G−F^ mice reduced AD-associated pathology and cognitive impairment.

## Discussion

In this study, we have identified a new role of the mammalian-specific MICOS component CHCHD6 in APP-associated AD pathology (Fig. 8d). Specifically, we observe circular feedback between CHCHD6 levels and APP processing. Under normal basal conditions, we observed that CHCHD6 and APP interact and stabilize each other. However, under AD-like pathological conditions of augmented amyloidogenesis, APP induces a selective loss of CHCHD6 at the transcriptional level through binding of the AICD–Fe65–Tip60 complex to the CHCHD6 promoter. Downregulation of CHCHD6 in AD models then reduces the amount of bound APP-CHCHD6, which promotes accumulation of APP on the MAM for amyloidogenic processing. More AICD is then generated due to enhanced APP processing, which in turn magnifies transcriptional repression of CHCHD6. This circular feedback between CHCHD6 and APP thus amplifies AD pathogenic signals, resulting in accelerated Aβ accumulation, cognitive deficits, and neurodegeneration in AD. Importantly, this pathologic feedback loop is restrained when levels of CHCHD6 are therapeutically elevated.

APP is a transmembrane protein that contains an endoplasmic reticulum (ER) leader sequence followed by a cryptic mitochondrial targeting peptide, allowing its localization on either ER or mitochondria [32, 71]. APP can be transmembrane-arrested on the mitochondria, forming a stable complex with mitochondrial protein importation machinery, such as translocase of the outer membrane (TOM) and translocase of the inner membrane (TIM). These translocation intermediate complexes are toxic and disrupt mitochondrial import, and are associated with mitochondria depolarization, increased ROS production, reduced cytochrome oxidase activity, and impaired ATP synthesis [13, 32, 43]. Furthermore, mitochondrial morphology and dynamics can be influenced by mitochondrial APP accumulation in APP-overexpressing mice, as well as in fibroblasts from AD patients, which results in elevated mitochondria fragmentation and cellular dysfunction [67, 68]. Interestingly, we found that APP-induced mitochondrial phenotypes were similar to those caused by disruption of the MICOS complex, suggesting a potential molecular overlap between APP abnormality and MICOS complex dysregulation. Indeed, our results showed that either overexpression of APP WT or the presence of APP mutants inhibited CHCHD6 at the transcriptional level, which resulted in MICOS complex disassembly. This then led to mitochondrial bioenergetic failure, cytochrome c release, and neuronal loss. The degree of CHCHD6 decrease and related MICOS complex loss appear to depend on the severity of APP pathology. Thus, our findings provide a new line of evidence to support the pathological interaction between APP and mitochondrial dysregulation in the development and progression of AD.

Although the APP proteolytic product AICD can be rapidly degraded after generation through APP processing, it is stabilized by cofactors, like Fe65, and accompanied by transcription factors, like Tip60, to regulate genes that are important for neurite development and axonal transport [42]. Some of these same genes also contribute to early pathology in AD [8, 33, 74]. Here, we have identified CHCHD6 as a previously unrecognized target of AICD-mediated gene expression regulation. We observed that AICD binds to the CHCHD6 promoter to repress transcription of CHCHD6. Moreover, AICD-mediated CHCHD6 gene repression is consistent with the notion that loss of CHCHD6 and related MICOS loss are early molecular events of AD pathogenesis prior to Aβ accumulation and cognitive deficits. We have also demonstrated that neuronal downregulation of CHCHD6 accelerates development of AD neuropathology, whereas neuronal compensation for CHCHD6 loss mitigates AD-like pathology and cognitive deficits. Thus, our findings establish a direct interaction between the amyloidogenic pathway and MICOS complex impairment in the pathophysiology of AD. In the current study, we mainly used APP mutant-expressing neuronal cells and mouse lines that are associated with familial AD. However, our data also showed that either overexpression or downregulation of APP WT inversely affected CHCHD6 protein level. CHCHD6 protein levels and immunodensity were also consistently decreased in the hippocampus of sporadic AD patients. Thus, when the amyloidogenic pathway is augmented in either familial or sporadic AD, CHCHD6 deficiency could result in MICOS disassembly, mitochondrial bioenergetic failure, cholesterol accumulation, neuropathology and cognitive deficiency in AD.

Brain cholesterol accumulation is a pathological feature of AD that causes region-specific loss of synapses [48] and increased Aβ production and Aβ toxicity [38]. As for now, the molecular mechanism through which CHCHD6 affects cholesterol homeostasis is incompletely understood. Our results, for the first time, showed a regulatory effect of CHCHD6 on the expression of genes involved in cholesterol metabolism. Two genes that are significantly influenced by CHCHD6 deficiency are *hmgcr* and *cyp46a1*. This further underscores CHCHD6 as a potential therapeutic target of AD, the manipulation of which acts on cholesterol biosynthesis and elimination simultaneously. Moreover, neuron-specific compensation of CHCHD6 may avoid the side effect of cholesterol synthesis inhibitors, like statins, in regulating cholesterol levels in plasma and peripheral tissue. Our recent study proposed a model in which CYP46A1 expression is suppressed by ATAD3A oligomerization under AD-associated conditions [76]. Given that both CHCHD6 and ATAD3A localize on the mitochondrial contact site [14, 76, 77], this could potentially explain the observed phenotypes. Future study of CHCHD6-ATAD3A interaction will help to test this possibility and further address the impact of CHCHD6 on cholesterol turnover. Thus, it remains to be determined whether the CHCHD6-mediated effects on neuronal cholesterol occur independently or in conjunction with mitochondria. We also note that familial and sporadic AD subjects display increased cholesterol esters in lipid raft-like MAMs enriched in cholesterol and sphingolipids [4]. Amyloidogenic processing of APP proteolytic fragment C99 takes place in lipid rafts of the MAM, where γ-secretase activity is enriched. C99 on the MAMs impairs cellular lipid homeostasis and alters membrane lipid composition in AD [37, 45], which further facilitates Aβ production. Thus, it is also possible that CHCHD6 deficiency indirectly elicits neuronal cholesterol accumulation by promoting C99 accumulation on the MAMs.

Aberrant cristae architecture and diminished mitochondrial respiratory capacity are common pathological signatures observed across many human diseases. Current studies of the association of MICOS with human diseases have been mainly focused on mitochondrial dysfunction. Deletion or mutation of a specific MICOS component in a particular disease disrupts the cristae structure and impairs mitochondrial function. The findings obtained from the present study unravel a unique function of a MICOS subunit, CHCHD6, in regulating APP amyloidogenic processing (one of the hallmarks of AD) and neuronal cholesterol accumulation (a known risk factor of AD). Our study indicates that pathological deficiency of CHCHD6 is an inducer that acts upstream of both APP amyloidogenic processing and neural cholesterol accumulation, which provides a direct mechanistic explanation for the impact of mitochondrial damage on AD neuropathology. This is supported by our observation that compensation for the loss of CHCHD6 restored cholesterol homeostasis, suppressed amyloidogenesis, and reduced neurodegeneration and cognitive deficits in AD models. Therefore, our findings uncover previously unknown insights into AD pathogenesis and demonstrate that CHCHD6 loss-of-function is a potential therapeutic target for AD.

## Supplementary Information

Below is the link to the electronic supplementary material.

Supplementary file1 (PDF 8063 KB)
