# Supplementary material for: A CHCHD6–APP axis connects amyloid and mitochondrial pathology in Alzheimer’s disease
Source: Acta Neuropathol. 2022 Sep 14;144(5):911–38. doi: 10.1007/s00401-022-02499-0 (PMC9547808; doi:10.1007/s00401-022-02499-0)

The CHCHD6-APP axis connects amyloid and mitochondrial pathology in Alzheimer's disease

Yutong Shang[1], Xiaoyan Sun[1], Xiaoqin Chen[1], Quanqiu Wang[2], Evan J Wang [2,3], Emiko Miller[4,5], Rong Xu[2], Andrew A Pieper[4,5] and Xin Qi[1,*]

[1]Department of Physiology & Biophysics, Case Western Reserve University School of Medicine, Cleveland, OH 44106, USA; [2]Center for Artificial Intelligence in Drug Discovery, Case Western Reserve University School of Medicine, Cleveland, OH 44106, USA; [3]Beachwood High School, Beachwood, OH 44122, USA; [4]Harrington Discovery Institute, University Hospitals Cleveland Medical Center, Cleveland, OH 44106 USA; [5]Department of Psychiatry Case Western Reserve University, Geriatric Research Education and Clinical Centers, Louis Stokes Cleveland VAMC, Cleveland, OH 44106 USA

***Corresponding author:**
Xin Qi Ph.D., Department of Physiology and Biophysics, Case Western Reserve University School of Medicine, 10900 Euclid Ave, E516, Cleveland, Ohio, 44106-4970, USA. Tel: 216-368-4459; Fax: 216-368-5586; E-mail: xxq38@case.edu

Supplementary Figure Legends

Supplementary Figure 1. CHCHD6 selectively decreases in APP-related AD models. (a) BN-PAGE analysis of MICOS complex in stable APP Neuro2a cells was performed with anti-Mitofilin antibody. Histogram: relative density of Mitofilin-immunoreactive band around 720 kDa to ATPB. WT: n =6; APP^{wt}- and APP^{swe}: n=9. (b) Mitochondrial fractions were isolated from hippocampus of WT, APP^{NL-G-F} and APP^{NL-F} mice at the ages of 3, 6, and 9 months and subjected to BN-PAGE analysis. Histogram: relative density of Mitofilin-immunoreactive band around 720 kDa to ATPB. n = 3 mice/group. (c) RNA was extracted from the hippocampus of 6-month-old 5XFAD mice and age-matched WT mice. The expression of MICOS complex components were analyzed by qPCR. Heat map analysis shows the mean of the genes analyzed. *, $p < 0.05$ (WT mice vs. 5XFAD mice). Mitofilin, CHCHD3, CHCHD6: n=5; APOO, MINOS1, QIL1, APOOL: n=8. All data are shown as mean ± SEM. Data were compared using one-way ANOVA with Tukey's post hoc test in a, b, and unpaired Student's t-test in c.

Supplementary Figure 2. CHCHD6 mainly expresses in neurons and decreases in APP-related AD mice and AD patient postmortem brains. (a) Brain sections from 3, 6, and 9 months of WT and 5XFAD mice were stained with anti-CHCHD6 and anti-6E10 antibodies. The CHCHD6 and 6E10 immunodensities (n = 3 mice/group) were quantified and shown in b. (c) Brain sections from 6-month-old WT and APP^{NL-G-F} mice were stained with anti-CHCHD6 and anti-Iba1 antibodies (left). Brain sections from 6-month-old WT and 5XFAD mice were stained with anti-CHCHD6 and anti-GFAP antibodies (right). (d) Postmortem cortex sections from control subjects and AD patients were stained with anti-CHCHD6 and anti-NeuN antibodies (n =5 individuals/group). The intensity of CHCHD6 in $NeuN^+$ cells was quantified. DAPI was used to label nuclei. The data are presented as mean ± SEM. The data in panel b were compared by two-way ANOVA with Tukey's post-hoc test, and the data in panels d were compared by the unpaired Student's t-test.

Supplementary Figure 3. CHCHD6 is associated with APP and AD. Neuro2a cells were treated with oligomeric $A\beta_{1-42}$ peptides (5 μM) at the indicated times. (a) Total protein levels of MICOS components were subjected to WB analysis with the indicated antibodies. (b) The expression of CHCHD6, Mitofilin and CHCHD3 were analyzed by qPCR. (c) The integrated gene-pathway-phenotype network with labeled data resources was used to analyze the relationship between CHCHD6 and AD (see method). All the nodes are standardized using standard biomedical terminologies, with each node representing a unique biomedical entity. (d) Top 10 ranked genes that are highly associated with CHCHD6 within a total of 30,049 prioritized genes. (e) Top 10 ranked pathways and AD-related

human phenotypes that are associated with CHCHD6. (f) Stable CHCHD6 KO HT-22 cells was generated by CRISPR-cas9. CHCHD6 knockout efficiency was examined by WB. (g) Represent pictures of PLA assays of APP^{NL-G-F} mice. Enlarged pictures are shown in Fig 3d. (h) Brain sections from 6-month-old WT and 5XFAD mice (n = 4 mice/ group) were stained with anti-APP and anti-CHCHD6 antibodies, and subjected to PLA analysis. Histogram: the number of PLA-positive puncta (red) was quantified from at least four separate fields of each sample. Representative blots from at least 3 independent experiments are shown. The data in panels a-b were compared using one-way ANOVA with Tukey's post hoc test, and the data in panels h were compared by unpaired Student's t-test.

Supplementary Figure 4. CHCHD6 deficiency induces mitochondrial damage. (a) Mitochondrial fractionations were performed with control and CHCHD6 KO HT-22 cells and WB was carried out with the indicated antibodies. The intensities of C99 and APP on the MAM fractions were quantified and shown in histograms. n=3 independent experiments. (b) Represent pictures of PLA assays of APP^{NL-G-F} mice. Enlarged pictures are shown in Fig 3h. (c) Brain sections from 6-month-old WT and 5XFAD mice (n = 4 mice/ group) were stained with anti-APP and anti-FACL4 antibodies, and subjected to PLA analysis. Histogram: the number of PLA-positive puncta (red) was quantified from at least four separate fields of each sample. Representative blots from at least 3 independent experiments are shown. (d) Control and CHCHD6 KO HT-22 cells were stained with anti-LC3B antibody to assess autophagy. The size and number of LC3B puncta was quantitated and shown in the histogram. At least 50 cells/group were analyzed, and the data were from 3 independent experiments. Representative images are shown on the top. (e) Mitochondrial lysates were harvested from control and CHCHD6 KO HT-22 cells and WB was performed with the indicated antibodies. The data are presented as the mean ± SEM. The data were compared by the unpaired Student's t-test**.**

Supplementary Figure 5. CHCHD6 deficiency induces neuronal cholesterol accumulation in AD models. (a) Timeline of AAV injection in APP^{NL-F} mice. AAV-Scramble shRNA-mCherry and AAV-CHCHD6 shRNA-mCherry were injected into bilateral hippocampus of 6-month-old mice. (b) Brain sections from 12-month-old AAV-Scramble shRNA or AAV-CHCHD6 shRNA-injected WT or APP^{NL-F} mice were stained with anti-CHCHD6 and anti-NeuN antibodies. Histogram: the CHCHD6 immunodensity in $NeuN^+$ cells (n = 3 mice/group) was quantified. Total brain lysates were harvested from the hippocampus of 12-month-old mice at the indicated groups. Levels of mitochondrial proteins, LonP, VDAC, Tim23 (c), and MICOS components, Mitofilin and CHCHD3 (d) were examined by WB. Histogram: the relative density of Mitofilin and CHCHD3 to actin. n =8 independent experiments. (e) mRNAs were extracted from control and CHCHD6 KO HT-22 cells. Genes involved in cholesterol metabolism were analyzed by qPCR (n=3-7 independent experiments). Heat map: the mean of the genes analyzed. *, $p < 0.05$; **, $p < 0.01$; ***, $p < 0.001$; ****, $p < 0.0001$ (HT-22 EV control vs. CHCHD6 KO cells). Representative images and blots from at least three independent experiments are shown. The data are presented as mean ± SEM. The data in panel b, d were compared by one-way ANOVA with Tukey's post-hoc test, and the data in panel e were compared by the unpaired Student's t-test.

Supplementary Figure 6. Viral vector-mediated downregulation of CHCHD6 accelerates cognitive deficits and AD pathology in APP^{NL-F} KI mice. (a) The Y-maze test was performed with 12-month-old mice (WT/Scramble shRNA: n = 20 mice; n = 22 mice/group for the WT/CHCHD6 shRNA, and APP^{NL-F}/Scramble shRNA, and APP^{NL-F}/CHCHD6 shRNA groups). (b) Body weights of mice at the indicated groups were recorded. (WT/Scramble shRNA: n = 21 mice; n = 24 mice for the WT/CHCHD6 shRNA; n = 23 mice/group for APP^{NL-F}/Scramble shRNA, and APP^{NL-F}/CHCHD6 shRNA groups). (c) Brain sections from the indicated groups were stained with anti-APP and anti-FACL4 followed by PLA. The positive PLA puncta (green) was quantified from at least four separate fields of each sample. n=4 mice/group. (d) Total brain lysates were harvested from the hippocampus of 12-month-old AAV-Scramble shRNA or AAV-CHCHD6 shRNA-injected WT or APP^{NL-F} mice. The levels of PSD95 and synaptophysin were analyzed by WB. Histogram: the relative density of PSD95 or synaptophysin to actin (n=6 mice/group for synaptophysin; n=8 mice/group for PSD95). Representative blots from at least three independent experiments are shown. The data are presented as the mean ± SEM. The data in panel a, c and d were compared by one-way ANOVA with Tukey's post-hoc test.

Supplementary Figure 7. Compensation for the loss of CHCHD6 reduces neuropathology and cognitive deficits in APP^{NL-G-F} KI mice. (a) Timeline of AAV injection in APP$^{NL-G--F}$ mice. AAV-CHCHD6-eGFP or AAV-EV-eGFP were injected into the bilateral hippocampus of 3-month-old mice. Total brain lysates were harvested from the hippocampus of 9-month-old AAV-CHCHD6 or AAV-EV-injected WT or APP^{NL-G-F} mice. The levels of indicated proteins were analyzed by WB. Histogram: the relative density of LonP, VDAC, Tim23 (b) (n=6 mice/group); MICOS components, Mitofilin and CHCHD3 (c) (n=6 mice/group) to actin. (d) The Y-maze test was performed with 9-month-old mice (WT/EV and APP^{NL-G-F}/CHCHD6 groups: n = 25 mice/group; n = 26 mice for WT/CHCHD6; and n = 24 mice for APP^{NL-G-F}/EV). (e) Body weights of mice of the indicated groups were recorded. (WT/EV and APP^{NL-G-F}/CHCHD6 groups: n = 25 mice/group; n = 27 mice for WT/CHCHD6; and n = 24 mice for APP^{NL-G-F}/EV). (f) Brain sections from the indicated groups were stained with anti-APP and anti-FACL4 followed by PLA. The positive PLA puncta (red) was quantified from at least four separate fields of each sample. n=3 for APP^{NL-G-F}/EV and n=4 mice/group for other groups. Representative blots from at least three independent experiments are shown. The data are presented as mean ± SEM. The data were compared by one-way ANOVA with Tukey's post-hoc test.

Supplementary Figure 8. Compensation for the loss of CHCHD6 increased the levels of synaptic proteins in APP^{NL-G-F} KI mice. Total brain lysates were harvested from the hippocampus of 9-month-old AAV-CHCHD6 or AAV-EV-injected WT or APP^{NL-G-F} mice. The levels of indicated proteins were analyzed by WB. Histogram: the relative density of PSD95 and synaptophysin (n=5 mice/group for synaptophysin and n=6 mice/group for PSD95). Representative blots from at least three independent experiments are shown. The data are presented as mean ± SEM. The data were compared by one-way ANOVA with Tukey's post-hoc test.

Supplementary Table 1: The information of antibodies used in the study

Antibody	Manufacture	Catalog #	Dilution
**ATPB**	ProteinTech (Rosemont, IL, USA)	17247-1-AP	1:2000
**CHCHD6**	ProteinTech (Rosemont, IL, USA)	20639-1-AP	1:2000
**CHCHD3**	ProteinTech (Rosemont, IL, USA)	25625-1-AP	1:1000
**Tom20**	ProteinTech (Rosemont, IL, USA)	11802-1-AP	1:4000
**Lonp**	ProteinTech (Rosemont, IL, USA)	15440-1-AP	1:1000
**FACL4**	Santa Cruz Biotechnology (Dallas, TX, USA)	sc-365230	1:1000
**c-Myc**	Santa Cruz Biotechnology (Dallas, TX, USA)	sc-40	1:1000
**Tim23**	Santa Cruz Biotechnology (Dallas, TX, USA)	sc-514463	1:1000
**APP**	Abcam (Cambridge, UK)	ab32136	1:5000
**cytochrome C**	Abcam (Cambridge, UK)	ab110325	1:10000
**VDAC**	Abcam (Cambridge, UK)	ab14734	1:2000
**Mitofilin**	Abcam (Cambridge, UK)	ab110329	1:500
**synaptophysin**	Abcam (Cambridge, UK)	ab32127	1:10000
**Fe65**	Abcam (Cambridge, UK)	ab5668	1:500
**C-terminal of APP (C99, C83)**	Sigma-Aldrich (St. Louis, MO, USA)	A8717	1:5000
**NeuN (A60)**	Sigma-Aldrich (St. Louis, MO, USA)	MAB377	1:1000
**FLAG**	Sigma-Aldrich (St. Louis, MO, USA)	F3165	1:2000
**β-actin**	Sigma-Aldrich (St. Louis, MO, USA)	A1978	1:10000
**Iba1**	Wako Chemicals (Japan)	019–19741	1:1000
**GFAP**	MilliporeSigma (Burlington, MA, USA)	MAB360	1:1000
**PSD-95 antibody**	Invitrogen (Waltham, MA, USA)	MA1-045	1:500
**anti-β-amyloid 1-16 (clone 6E10)**	BioLegend (San Diego, CA, USA)	803015	1:2000
**Tip60**	Cell Signaling (Danvers, MA, USA)	12058	1:200
**LC3B**	Cell Signaling (Danvers, MA, USA)	2775S	1:2000
**NeuN**	MilliporeSigma (Burlington, MA, USA)	ABN90	1:1000
**AICD**	BioLegend (San Diego, CA, USA)	811901	1:200
**SigmaR1**	ProteinTech (Rosemont, IL, USA)	15168-1-AP	1:1000
**p62**	ProteinTech (Rosemont, IL, USA)	18420-1-AP	1:1000

Supplementary Table 2: Primers used for qPCR

Gene name	Forwarded primer	Reverse primer
**Hu D6 F**	ACCAAGCACTCCAAGGCATC	GTGTCACGGCGTCTTAGCTC
**Hu mitofilin**	CGGGCCTGTCAGTTATCGG	CAATGGACGGAGGACAAACTT
**Hu D3**	GCAAGCCAAGAAAGAATCCGA	TTCCTCCTCGCTACATATCCTC
**Hu APOOL**	CCAAAAAGCAGCTAGTGAAACC	AGTTGCAGTGCGGATGGAA
**Hu APOO**	TCCTGAGGGTCAATCGAAGTAT	CTCGCAATAGTGTCGGAGC
**Hu QIL1**	TCAGCCAGTACGTGTGTCAG	GCCTGCATTCCAGGAGTCAC
**Hu MINOS1**	GATGCGGTCGTGAAGATAGGT	CCAGAACCGAAGGCTAATGG
**Ms D6**	TGTCTGAAAGTGTTGTGAACCG	GATGGCTGGTAGAGGGACAGT
**Ms mitofilin**	GGAGGGATTGGTGGCACTATC	TTTGAGCTGCCCCTGTAGATG
**Ms D3**	GCGGACGAGAACGAGAACAT	TCGCTGAGACTTAGAGCCAGA
**Ms APOOL**	ATGGCGGCCTTTAGGATGG	TCCGGTCTCACTAGCTGCT
**Ms APOO**	GGTTGTGGGGAGGATGAAGC	GCTGCATAGACTCTGAAGGTGA
**Ms QIL1**	GTGGTCGCTAATGAGGTTCCT	GCTGGCACACATATTGGCTG
**Ms MINOS1**	ACACGGTCGTGAAGCTAGGTA	CAGTTGGAGTAGGCCATTCCC
**Ms CHCHD10**	CAGCCGGGTCTTATGGCTC	CAGGCTCTGAATTTCCCCCAC
**Srebf2**	ATGATCACCCCGACGTTCAG	GGTCGCTGCGTTCTGGTATATC
**Hmgcr**	TTGGTCCTTGTTCACGCTCAT	TTCGTCCAGACCCAAGGAAAC
**Hmgcs2**	GGAAGCCTTTGGGGACGTTA	ACACTCCAACCCTCTTCCCT
**Ldlr**	ACCTGCCGACCTGATGAATTC	GCAGTCATGTTCACGGTCACA
**Lxr-alpha**	AGCGTCCATTCAGAGCAAGTG	CACTCGTGGACATCCCAGATCT
**Lxr-beta**	ACTCGGAGCAGGTCTTTGCAT	CCTACTCGTGCACATCCCAGAT
**ApoE**	GGCCCAGGAGAATCAATGAG	CCTGGCTGGATATGGATGTTG
**Abca1**	AGGCCGCACCATTATTTTGTC	GGCAATTCTGTCCCCAAGGAT
**CYP46A1**	TCCTCTCCTGTTCAGCACC	CAGCTTGGCCATGACAACT
**Lrp1**	ACTATGGATGCCCCTAAAACTTG	GCAATCTCTTTCACCGTCACA
**Apoa2**	CTGACCTGACAAGGGGTGTC	ATGGCAAAGATTTGGTGGAG
**Lipe**	CCTGTCTCGTTGCGTTTGTA	ACGCTACACAAAGGCTGCTT
**Snx17**	CAGGGGTCAAAGAGAACAGC	GTGAATGGAGTCCTGCACTG
**Prkaa1**	GTCAAAGCCGACCCAATGATA	CGTACACGCAAATAATAGGGGTT
**Ubc-7**	CTGGCAGAACTCAACAAAAATCC	AGATGAGCCTTAAAAACACCACC
**Gp78**	ACAAAGACCTATCTGAAACGTCC	AGGGAGCTTGTGGCTCAGTA
**Ms GAPDH**	GACTTCAACAGCAACTCCCAC	TCCACCACCCTGTTGCTGTA
**Hu GAPDH**	GCGAGATCCCTCCAAAATCAA	GTTCACACCCATGACGAACAT

Supplementary Table 3: Human postmortem brain samples

ID	Age	Gender	Brain region	Clinical Final diagnosis	Dementia	NFT Braak Stage	AD pathology	CERAD score	Cerebrovascular pathology
**HSB 4328**	67	F	Hippocampus	Unaffected control	No	0	No indication	Negative	No
**HSB 4431**	68	F	Hippocampus	Unaffected control	No	0	No indication	Negative	Minimal atherosclerosis in the basilar cerebral vasculature
**HSB 4494**	67	M	Hippocampus	Unaffected control	No	0	No indication	Negative	No
**S06428**	63	F	Hippocampus	Unaffected control	No	I	Rare to sparse non-neuritic neocortical amyloid plaques	Negative	Atherosclerosis and arteriosclerosis.
**S07396**	55	M	Hippocampus	Unaffected control	No	0	No indication	Negative	Mild arteriosclerosis.
**S08610**	78	F	Hippocampus	Unaffected control	No	0	Non-neuritic amyloid plaques, cerebral cortex.	Negative	Atherosclerosis and arteriosclerosis.
**HCT15HAU-19-01**	65	M	Hippocampus Cortex	Unaffected control	No	0	No indication	Negative	No
**HCT15HAM-19-01**	54	M	Hippocampus Cortex	Unaffected control	No	0	No indication	Negative	No
**HCT15HBY-19-01**	53	M	Hippocampus Cortex	Unaffected control	No	0	No indication	Negative	No
**HCT16HCA-19-01**	56	M	Hippocampus Cortex	Unaffected control	No	0	No indication	Negative	No
**HCT17HER-19-01**	53	M	Hippocampus Cortex	Unaffected control	No	0	No indication	Negative	Not specified
**HSB 4382**	74	F	Hippocampus	AD	Yes	IV-V	Frequent numbers of neuritic plaques; More neurofibrillary tangles; neuronal loss; gliosis	Frequent	Foamy macrophages associated with cortical vascular structures
**HSB 4666**	60	F	Hippocampus	AD, early onset AD	Yes	V	Frequent numbers of neuritic plaques and neurofibrillary tangles; no Lewy bodies.	Frequent	Minimal atherosclerosis in the basilar cerebral vasculature
**HSB 4788**	65	M	Hippocampus	AD, early onset AD	Yes	V	Numerous well-formed neuritic plaques and more neurofibrillary tangles; no Lewy bodies.	Frequent	Minimal atherosclerosis in the basilar cerebral vasculature
**S02528**	74	F	Hippocampus	AD	Yes	V	Present neuritic plaques and neurofibrillary tangles, amyloid angiopathy	Frequent	Atherosclerosis and arteriosclerosis.
**S04925**	79	F	Hippocampus	AD	Yes	V	Present neuritic plaques and neurofibrillary tangles, amyloid angiopathy	Frequent	Small vessel cerebrovascular disease with atherosclerosis
**S03740**	86	F	Hippocampus	AD	Yes	V	Present neuritic plaques and neurofibrillary tangles, severe amyloid angiopathy	Frequent	Small vessel cerebrovascular disease with atherosclerosis
**HBGT-19-01**	68	F	Hippocampus and Cortex	AD	Yes	IV-V	High AD neuropathological changes (A3, B3, C3)	Frequent	Not specified
**HBBD-19-01**	70	M	Hippocampus and Cortex	AD	Yes	V-VI	NF tangles and neuropil threads, amyloid accumulation, cortical atrophy, amyloid angiopathy,	Frequent	Arteriolosclerosis, diffuse, mild
**HBGS-19-01**	78	F	Hippocampus and Cortex	AD	Yes	V-VI	High AD neuropathological changes (A3, B3, C3)	Frequent	Not specified
**HBAX-19-01**	86	F	Hippocampus and Cortex	AD	Yes	VI	High AD neuropathological change (B3, C3), plaque stage, frequent	Frequent	Arteriolosclerosis, diffuse, mild to focally moderate

**HBGE-19-01**	88	M	Hippocampus and Cortex	AD	Yes	V-VI	High AD neuropathological changes (A3, B3, C3)	Frequent	Moderate small vessel ischemic disease, remote lacunar infarcts in parietal cortex and thalamus

NFT: neurofibrillary tangle, AD: Alzheimer's disease, CERAD: Consortium to establish a registry for Alzheimer's disease

Supplementary Figure 1

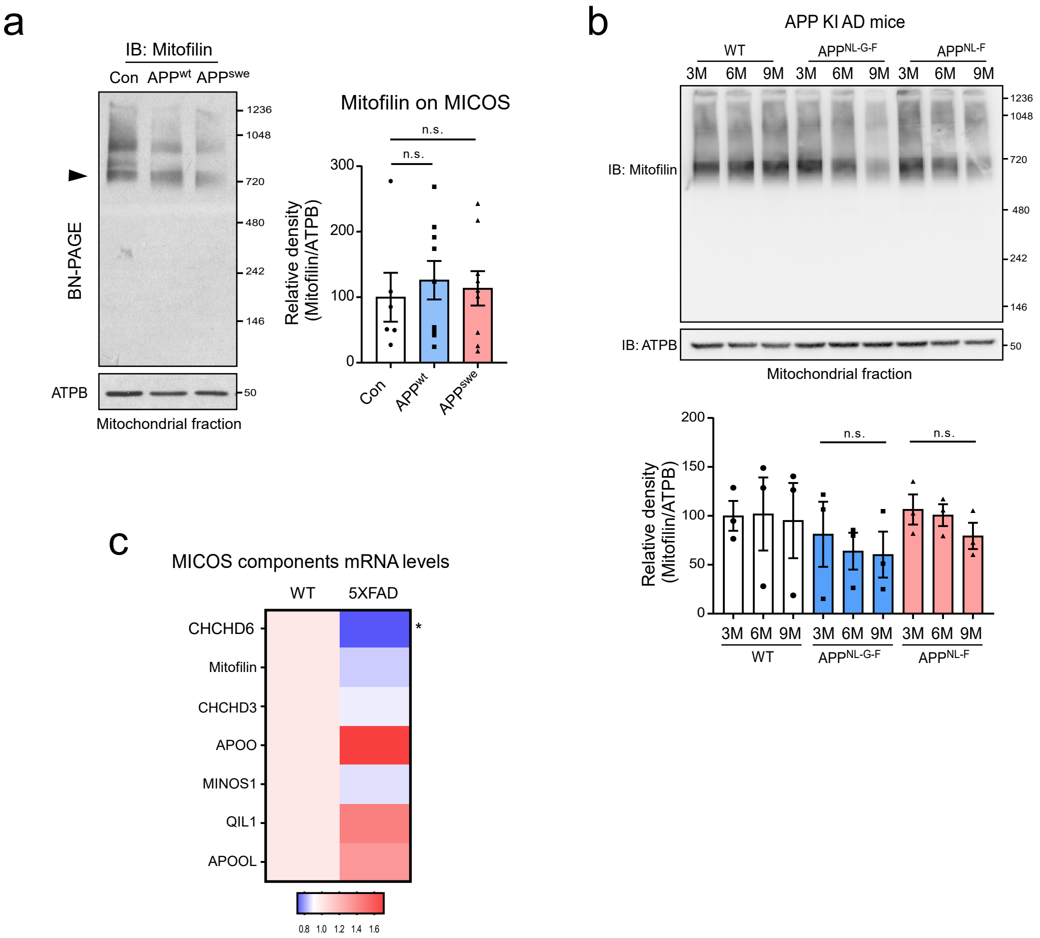

Supplementary Figure 2

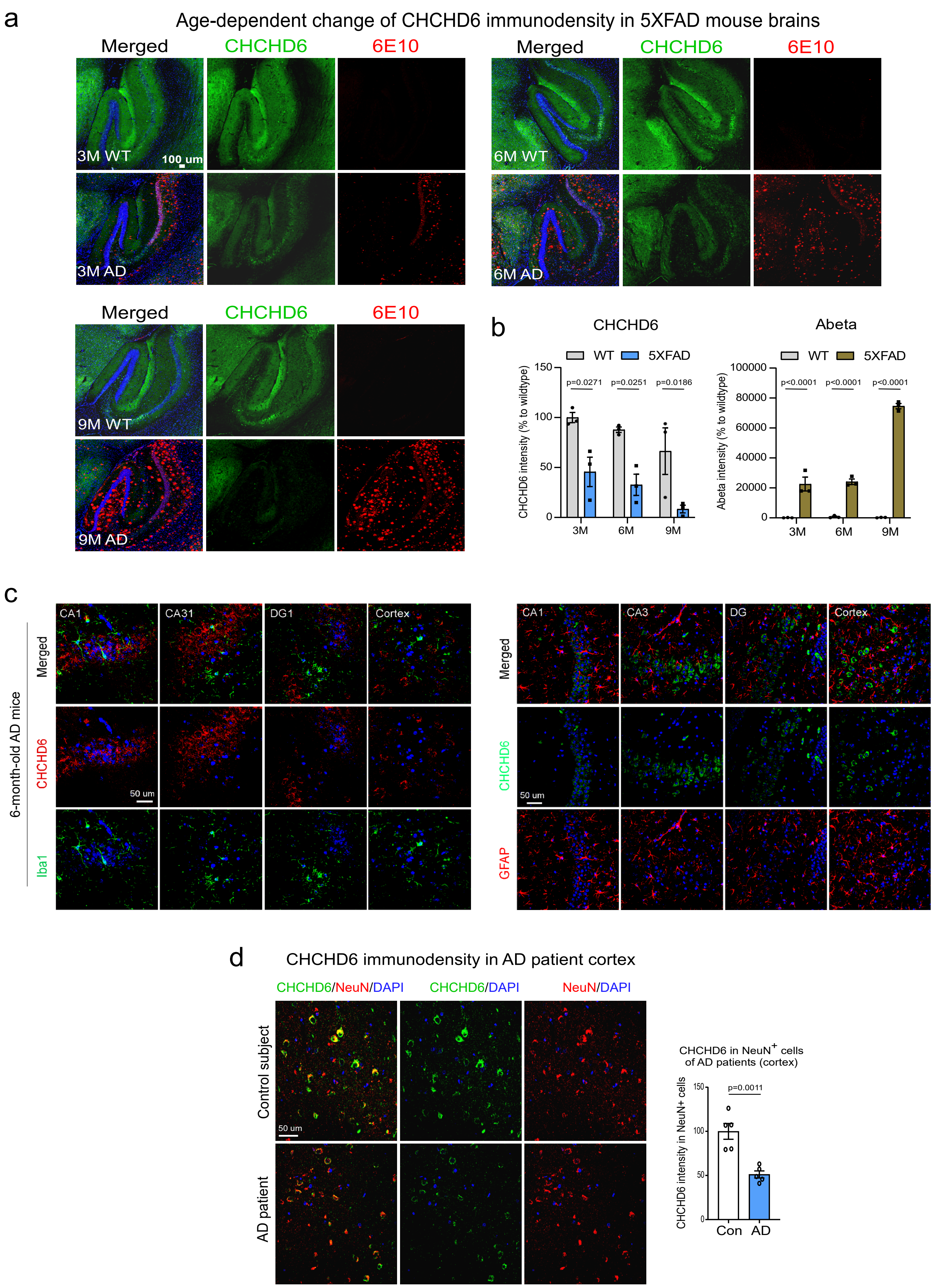

Supplementary Figure 3

a

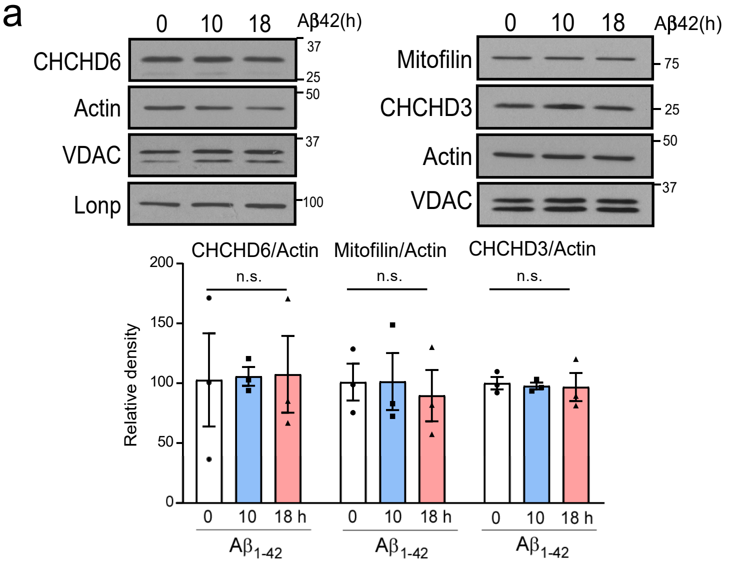

b

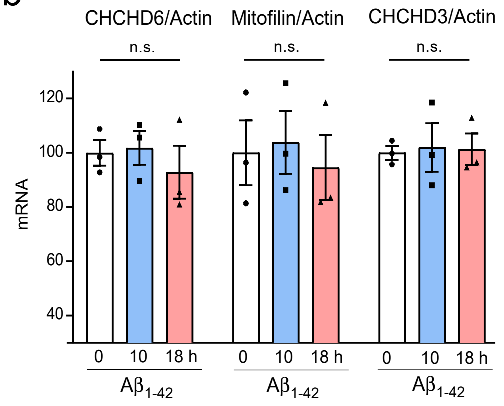

d

Top ranked predicted genes for the input "CHCHD6"

Gene	Rank (Top % among 30,049 genes)
APP	0.003
IMMT	0.010
SLC25A46	0.013
APOOL	0.017
MTX2	0.020
MMGT1	0.023
MYC	0.027
C19orf70	0.030
Tmed2	0.033
V APA	0.037
UBC	0.040
ERLIN1	0.043
GOLT1B	0.047
CHCHD2	0.050
HNRNPL	0.053
CD244	0.057
RAB7A	0.060
Rmdn3	0.063
PPP1CA	0.067
FBXO7	0.070

c

Numbers of nodes and edges on each subnetwork of GPPDN

Subnetwork	Nodes	Edges
Disease-Gene Network from OMIM (omimDGN)	5,983 diseases, 18,701 human genes	15,462
Disease-Gene Network from ClinVar (clinvarDGN)	5,842 diseases, 4,153 human genes	6,884
Disease-Gene Network (gwasDGN)	3,422 diseases, 18,532 human genes	98,218
Pathway-gene Network (PathGN)	8,868 human genes, 1,329 pathways	66,293
Gene-gene interaction network (PPIN)	22,982 human genes	382,256
Mouse Phenotype-gene network (PhenGN)	9,982 phenotypes, 11,021 genes	517,381
Chemical-Gene Network (ChemicalGN)	473,602 chemicals, 18,701 human genes	15,473,939

e

Top 10 ranked predicted pathways for the input "CHCHD6"

Pathway	Rank (Top% among 1,329 pathways)
Protein metabolism	0.075
Mitochondrial protein import	0.150
Natural killer cell mediated cytotoxicity	0.226
Immune System	0.602
GPCR signaling	0.677
Metabolism of lipids and lipoproteins	0.828
Alzheimer's disease	1.053
Tight Junction	1.129
Amyloids	1.580
Toll receptor cascades	1.655

Top 10 ranked predicted AD-related human phenotypes for the input "CHCHD6"

Name	Rank (Top % among 49,639 human diseases and phenotypes)
General cognitive ability	0.002
Ldl cholesterol x physical activity interaction	0.016
Neuropathy, hereditary motor and sensory	0.077
Gliosis	0.218
Cerebral amyloid angiopathy, app-related	0.268
App polymorphism	0.270
Alzheimer disease	0.276
Abnormal cholesterol homeostasis	0.391
Demyelination	0.401
Decreased brain weight	0.405

f

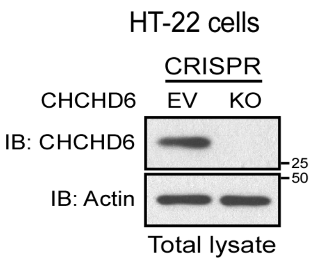

g

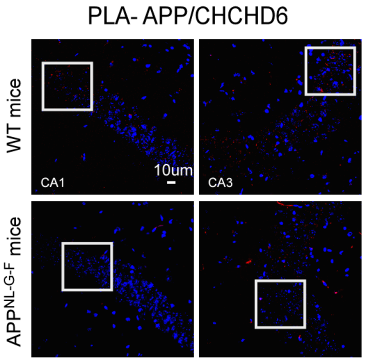

h

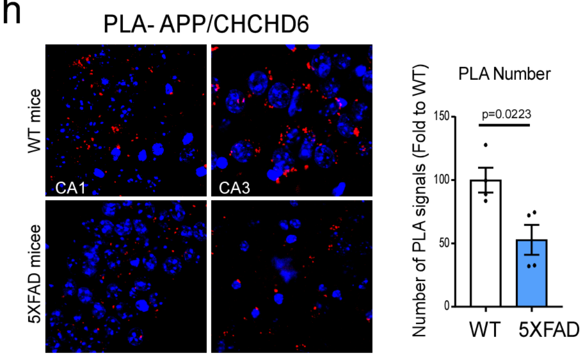

Supplementary Figure 4

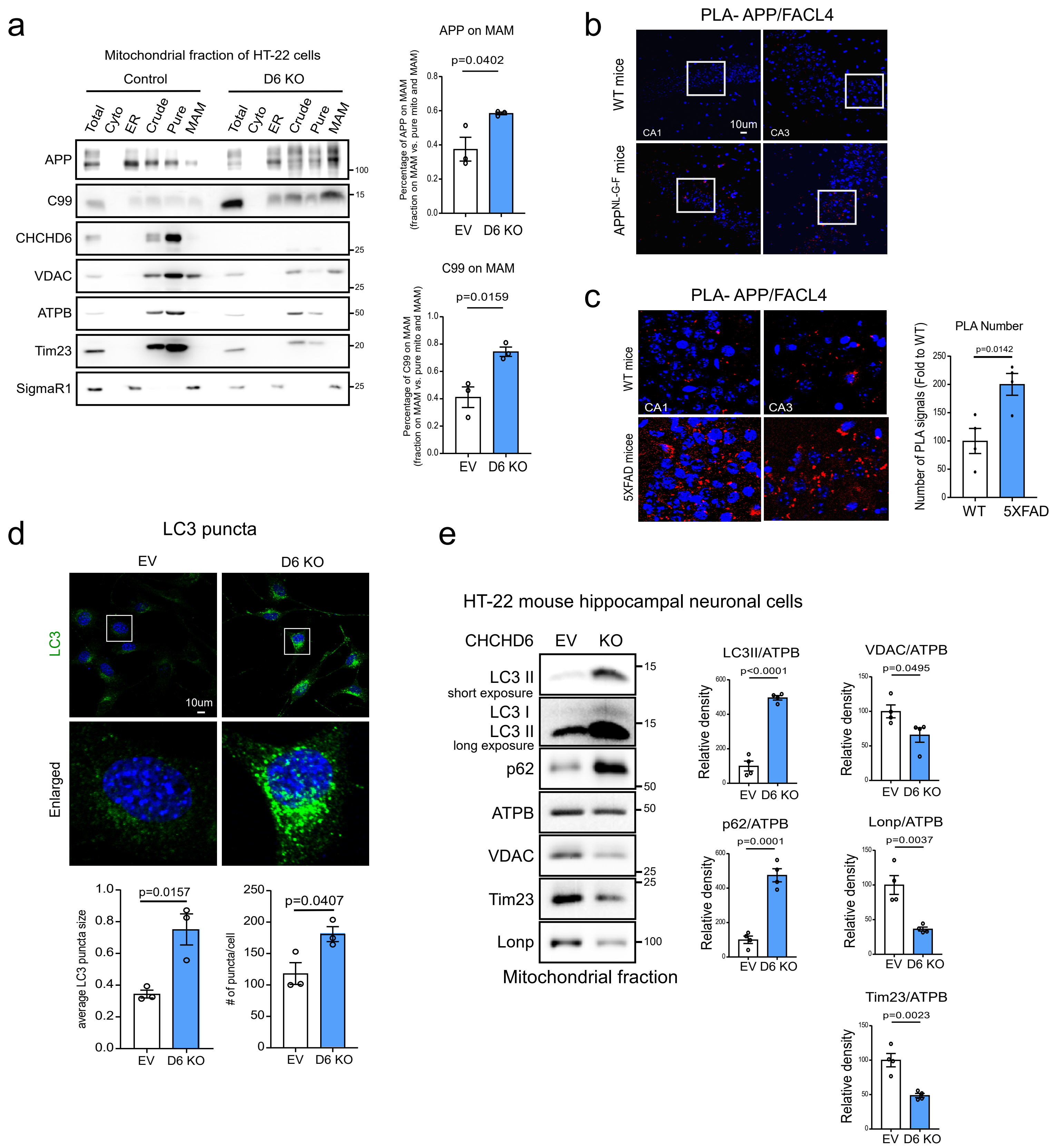

Supplementary Figure 5

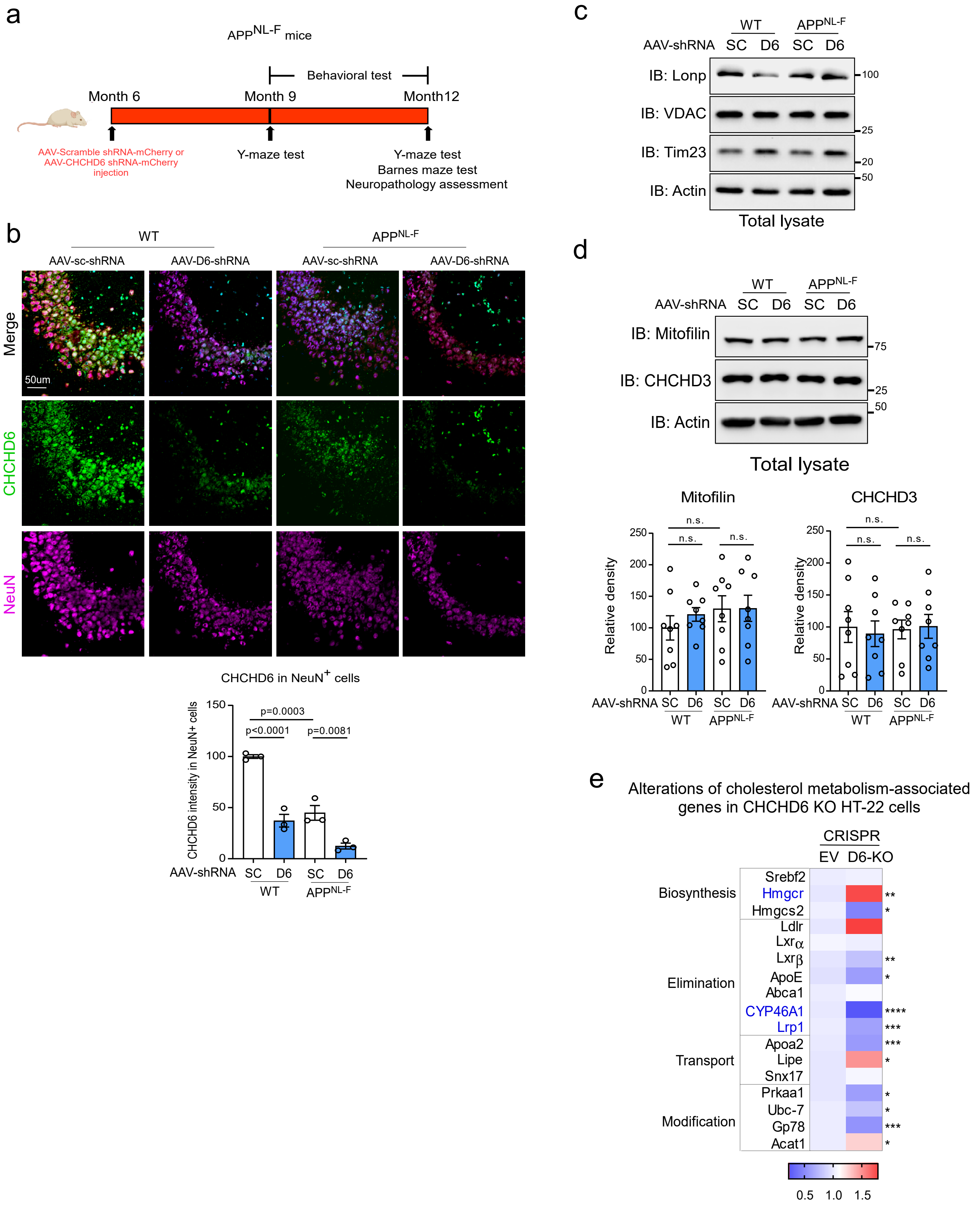

Supplementary Figure 6

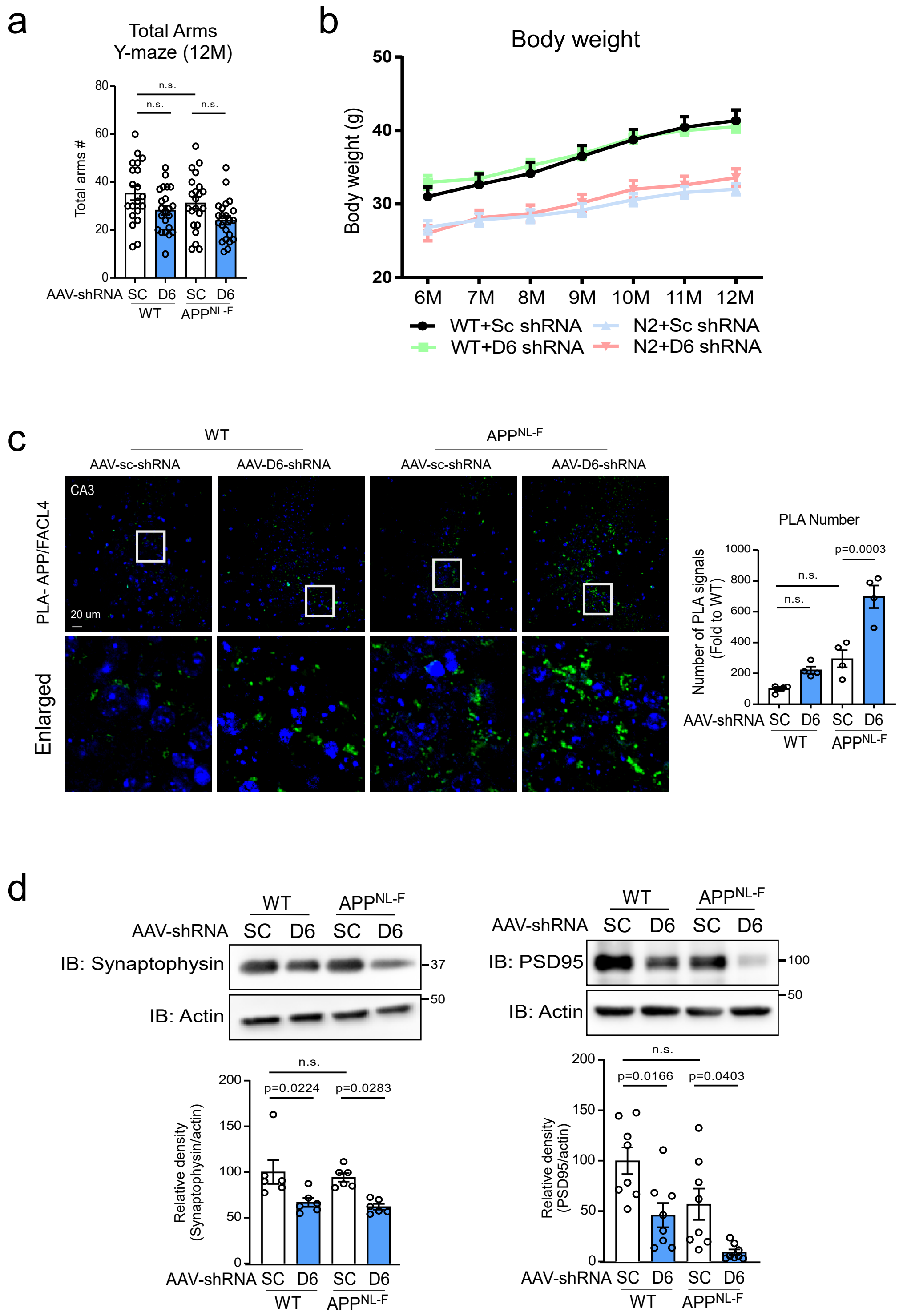

Supplementary Fig. 7

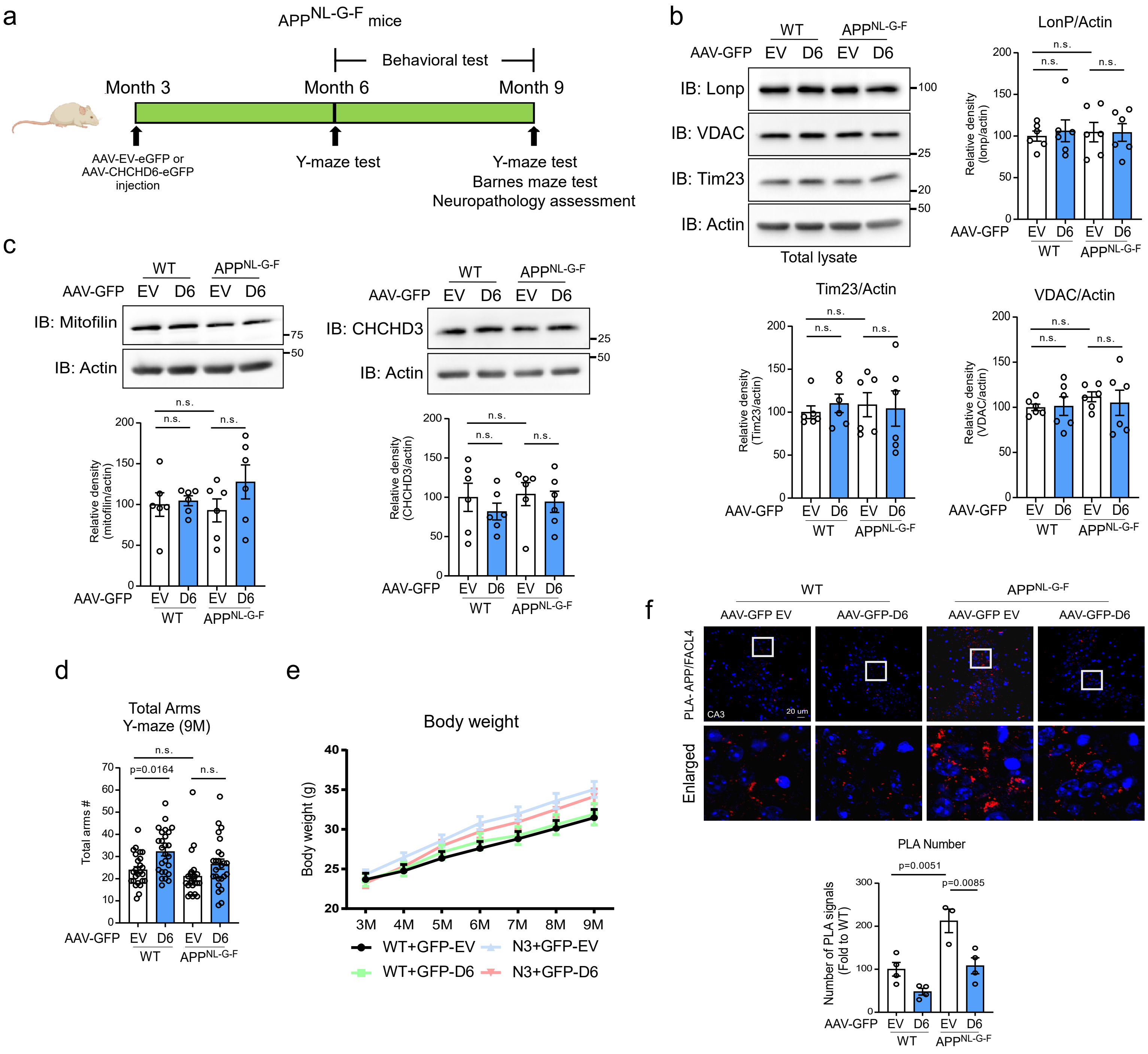

Supplementary Fig. 8

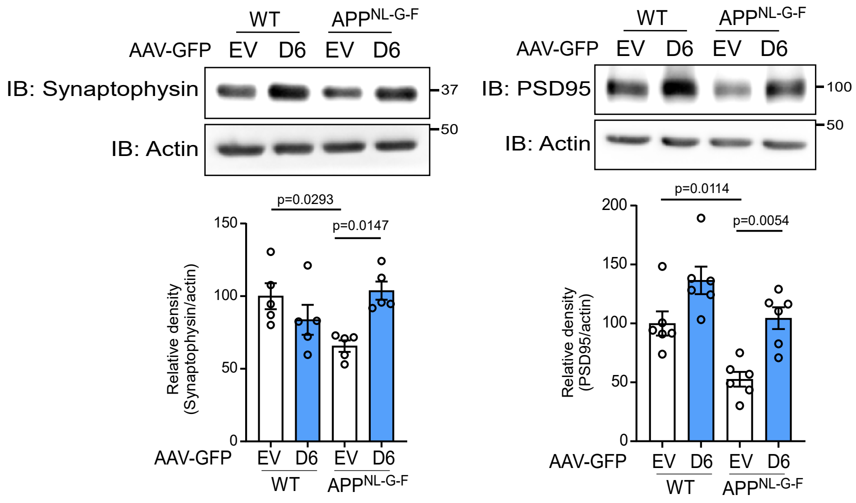

Supplement: Supplementary file 1 — Supplementary file1 (PDF 8063 KB) [file 401_2022_2499_MOESM1_ESM.pdf]
